# Simple SIR models with Markovian control

**DOI:** 10.1007/s42081-021-00107-1

**Published:** 2021-02-16

**Authors:** Krzysztof Bartoszek, Wojciech Bartoszek, Michał Krzemiński

**Affiliations:** 1 Department of Computer and Information Science, Linköping University, Linköping, 581 83 Sweden; 2 Department of Probability and Biomathematics, Gdańsk University of Technology, ul. Narutowicza 11/12, 80 233 Gdańsk, Poland

**Keywords:** SIR models, Markovian control, random dynamical systems, 47A35, 34F05, 37A30, 37H10, 60J05

## Abstract

We consider a random dynamical system, where the deterministic dynamics are driven by a finite-state space Markov chain. We provide a comprehensive introduction to the required mathematical apparatus and then turn to a special focus on the susceptible-infected-recovered epidemiological model with random steering. Through simulations we visualize the behaviour of the system and the effect of the high-frequency limit of the driving Markov chain. We formulate some questions and conjectures of a purely theoretical nature.

## Introduction

The spread of an epidemic and its related characteristics in a large population may be efficiently described by deterministic models. In particular, differential (or difference) equations are a popular first choice. However, if the population (or a particular subpopulation which plays a specific role in the epidemic) is small, then a stochastic approach is more appropriate. For example, at the initial stage of an epidemic when the disease is brought in from outside the considered population, only single individuals may be infected or we may even have the extreme case of a single individual (the so-called patient zero). Moreover, these infected individuals can be hidden from the health authorities. Our approach and results are general and can be also considered in epidemics amongst livestock (e.g. the recent spread of the African swine fever virus in Eastern Europe). We also do not focus on any particular mechanism of infection by the driving process. It can be due to transmission from natural interactions or even intentional contamination (from e.g. market competition). Alternatively, we may be in a situation, where the disease spread is impossible to observe. This is, in particular, true with the current dynamics of the SARS-CoV-2 virus. What is observed is the number of confirmed cases (based on tests) and confirmed case fatalities. However, there is a very large number of asymptomatic cases that are not discovered (Bai et al. 2020). These people are contagious and this is where the dynamics of the virus are taking place. However, we can only model, e.g. via SIR (susceptible-infected-recovered) systems, those parts of the population for which we have data. Therefore, it might be more appropriate to consider an IR, random dynamical system (RDS) model (for an overview of RDS with multiple examples see e.g. Smith and Thieme 2011; Ye et al. 2016; Ye and Qian 2019). The Infected dynamics are controlled by a random Markov process—i.e. from a randomly behaving population, we obtain observed infected individuals. There are two arguments for considering the unobserved dynamics to be random. Firstly, despite the population being large, various official or unofficial (e.g. self-isolation) mitigation strategies could result in random effects. This can be due to the randomness (from the perspective of the observer) in the moment of implementation, their scale, and also to what extent they are carried out by the population. Secondly, as one only tests a certain (small) part of the population and not completely at random but according to some protocol (e.g. only symptomatic, people who had contact with confirmed cases, risk groups, medical personnel, etc.), then one can expect randomness in the response—infected people randomly entered the particularly tested group. This is even true for the testing of symptomatic individuals—the symptoms of COVID-19 are consistent with many other illnesses, like the common cold or the flu. In all these situations the ignition points of the infection are limited in numbers. Therefore, one should not ignore all mathematical conditions of differential (smooth) models, like SI, SIR, etc. In this work we try to merge deterministic and stochastic methods in order to obtain a combined, hybrid model. We follow the approach of dividing the whole population into subgroups, arriving at a typical compartmental model. We assume that the total size of considered population is fixed and does not change through time. After normalization, we assume it to be simply 1. We follow standard nomenclature and call the healthy members who are exposed to the disease as susceptible (the total amount of the exposed is *S*), those who are *infected* are denoted by *I*, and finally, the “absorbing” group, the *recovered* (or removed from the population) is denoted by *R*. We have that $S + I + R = N$, where (in general) $N \equiv \mathrm {const}$, does not depend on time, and as already mentioned we take $N=1$.

The main aim of our paper is to illustrate the dependence of the quantitative analysis of an epidemic on a random steering process (which could represent the intensity of social relations). First in Sect. 2 we introduce the necessary mathematical background and our main results. Then in Sect. 3 we present two types of simulations. One, a toy model of a linear random dynamical system, and then a simple application, based on the SIR model to epidemiological data. In Sects. 4 and 5 we give a careful treatment of SIS and SIR models with our introduced random control mechanism We end our work with some some historical and outlook remarks, Sect. 6.

## Mathematical preliminaries, notation and results

The SIR model is in fact a flow $(S(t), I(t), R(t)) \in [0, N]\times [0,N]\times [0,N]$, governed by the system of differential equations. We wish to consider a wider setting by introducing general dynamical systems (cf. Smith and Thieme (2011)).

### Definition 1

Let $(X , \varrho )$ be a separable metric space. A map $\varPhi :[0, \infty )\times X \mapsto X$ is called a *semiflow * if

$$\begin{aligned} \begin{aligned} & (1) \ \ \varPhi (0, x) = x \ \ \text {for all } x\in X, \\ & (2) \ \ \varPhi (t+s , x) = \varPhi (t, \varPhi (s,x)) = \varPhi (s, \varPhi (t, x )) \ \ \text {for all } s, t \ge 0 \ \text {and all } \ x\in X. \end{aligned} \end{aligned}$$

We shall write $\varPhi _t(x)$ instead of $\varPhi (t,x)$, so we obtain

$$\begin{aligned} \varPhi _{t+s} = \varPhi _t\circ \varPhi _s = \varPhi _s \circ \varPhi _t .\ \end{aligned}$$

$\{\varPhi _t:t\ge 0\}$ is a semigroup, i.e. a representation of the semigroup $\mathbb {R}_+$ as transformations of *X*.

### Definition 2

A semiflow $\varPhi$ is called *state continuous* if for every $t \in [0,\infty )$ the mapping $\varPhi _t :X \mapsto X$ is continuous. $\varPhi$ is called *uniformly continuous* if for every $\varepsilon > 0$ there exists $\delta > 0$, such that if $\varrho (x,y) < \delta$, then

$$\begin{aligned} \sup \limits _{t\ge 0} \varrho (\varPhi _t(x) , \varPhi _t(y)) < \varepsilon \ . \end{aligned}$$

$\varPhi$ is called *uniformly continuous in finite horizon time * if for every $T > 0$ and all $\varepsilon > 0$ there exists a $\delta _T > 0$, such that if $\varrho (x,y) < \delta _T$, then

$$\begin{aligned} \sup \limits _{t\in [0, T]} \varrho (\varPhi _t(x) , \varPhi _t(y)) < \varepsilon \ . \end{aligned}$$

If for every fixed $x\in X$, the mapping

$$\begin{aligned}{}[0,\infty ) \ni t \mapsto \varPhi _t(x) \in X \ \ \ \text {is \ continuous}\ , \end{aligned}$$

then $\varPhi$ is called *time continuous*. Time continuous semiflows are called *1-Lipschitz in finite horizon time* if for every $T > 0$, there exists a constant $L = L_T \ge 0$ such that for all $x\in X$, all $t \in [0, T]$, and all $h \ge 0$ we have

$$\begin{aligned} \ \ \ \ \ \ \ \ \varrho (\varPhi _{t+h}(x) , \varPhi _t(x) ) \le L h \ .\end{aligned}\quad\quad \mathfrak {L} $$

Next we will need bounds on the expansiveness of $\varPhi _{t}$. For this we introduce a modulus function.

### Definition 3

Let $\varDelta :\mathbb {R}_+ \mapsto \mathbb {R}_+$ be a nondecreasing (growth) function satisfying $\varDelta (0) = 1$. We say that a semiflow $\varPhi$ is $\varDelta -$*equicontinuous* on *X* if for every pair $x , y \in X$ and all $t \in \mathbb {R}_+$ we have

$$\begin{aligned} \varrho (\varPhi _t(x) , \varPhi _t(y)) \le \varDelta (t)\varrho (x, y) \ . \end{aligned}\quad\quad \bigstar $$

In particular, $\varDelta$-equicontinuous semiflows are uniformly continuous in a finite horizon time. The function $\varDelta$ is said to be sub-multiplicative if for all $u_1, u_2, \ldots , u_k \ge 0$ we have

$$\begin{aligned} \varDelta (u_1)\varDelta (u_2)\cdots \varDelta (u_k) \le C \varDelta (u_1 + u_2 + \cdots + u_k). \end{aligned}\quad\quad \bigstar _{M}$$

Clearly, if $\varDelta (t) = \mathrm{e}^{\alpha t}$ for some $\alpha > 0$, then it satisfies ([Disp-formula Equ3]). It can be easily proved that condition ([Disp-formula Equ3]), with the extra assumption that $\varDelta$ is differentiable, reduces $\varDelta$ to the set of functions of the form $C\mathrm{e}^{\alpha t}$.

Notice that $\varDelta$-equicontinuity and the Lipschitz condition are related in the sense that as long as $\sup _{x\in X}\varrho (\varPhi _h(x), x) \le \widetilde{L} h$, then $\varrho (\varPhi _{t+h}(x), \varPhi _t(x)) = \varrho (\varPhi _t(\varPhi _h (x)), \varPhi _t(x) )\le \varDelta (t) \varrho (\varPhi _h(x), x) \le \varDelta (t)\widetilde{L} h$.

### Example 1

The classical example of a semiflow is an ordinary differential equation (ODE) system on $\mathbb {R}^{d}$. If the function $f(t,\mathbf {x}):[0,T]\times X \rightarrow \mathbb {R}^{d}$, where $X\subset \mathbb {R}^{d}$, is the solution of an ODE, then the associated semiflow is just $\varPhi _t(\mathbf {x})=f(t,\mathbf {x})$.

### Example 2

Let us consider a simple ordinary differential equation $f'(t) = \alpha (f(t)) f(t)$, where $f : [0, \infty ) \mapsto \mathbb {R}_+$ and $\alpha : [0, \infty ) \mapsto \mathbb {R}_+$ is an arbitrary positive, nonincreasing and continuous function. Its solutions (depending on the initial conditions $x\in \mathbb {R}_+$) constitute a semiflow $\varPhi _t(x) = f(t, x)$ on $\mathbb {R}_+$. Clearly the solutions satisfy

$$\begin{aligned} f(t,x ) = x\mathrm{e}^{\int _0^t \alpha (f(u,x)) \mathrm{d}u} \ . \end{aligned}$$

We can notice that $\varPhi _t(y) \ge \varPhi _t(x)$ if $0 \le x \le y$. It follows that $0 \le \alpha (f(u, y)) \le \alpha (f(u, x))$ if $0 \le x \le y$. Given that $0 \le x \le y \in \mathbb {R}_+$ we have

$$\begin{aligned} \begin{aligned} |\varPhi _t(y) - \varPhi _t(x) |&= | y\mathrm{e}^{\int _0^t \alpha (f(u,y)) \mathrm{d}u} - x\mathrm{e}^{\int _0^t \alpha (f(u,x)) \mathrm{d}u} | \\&\le |y - x| \mathrm{e}^{\int _0^t \alpha (f(u,x)) \mathrm{d}u} \le |y - x|\mathrm{e}^{\alpha (0) t} \ . \end{aligned} \end{aligned}$$

Our semiflow on the phase space satisfies condition ($\bigstar _{M}$).

### Example 3

The SIR model and its variations (SIS, SIRS, SEIR, SEIS Capasso 2008, but see also Sects. 4 and 5 for details) is described by a system of ODEs. For example the SIS model has a general form $S'(t)=f(t,S,I)$, $I'(t)=g(t,S,I)$, assuming that for all *t*, $S(t)+I(t)=1\equiv \mathrm {const}.$ Thus, the SIS model can be considered as a semiflow $\varPhi _t$ corresponding to those ODEs, where $X=[0,1]\times [0,1]$, $\varPhi _t((x,1-x))=(S(t,x),I(t,x))$.

Similarly, the SIR model ODE system defines a semiflow on the space $X=[0,1]^3$, $\varPhi _t((x,y,1-x-y))=(S(t,x),I(t,y),R(t,1-x-y))$, where for all $t\ge 0$, $S(t,x)+I(t,y)+R(t,1-x-y)=1$.

### Example 4

- Take $X = \mathbb {R}$ with the standard distance function $| \cdot |$ and define $\varPhi (t, x, v ) = \varPhi _t^{v} (x) = x + vt$. Clearly, they form the classical affine flow.
- Take $X = \mathbb {R}^2$ with the standard Euclidean distance and $\varPhi (t, (x,v), a ) = \varPhi ^a(t, (x,v)) = (a\frac{t^2}{2} + vt + x, at +v)$. This is another simple flow example.

We can see in all of the presented above examples that the transformations $\varPhi _t$ depend on some parameters. Our extensions concern these parameters. Given a nonempty set of parameters, $\varTheta$, we shall consider families of semiflows $\mathfrak {R }(\varTheta ) = \{ \varPhi ^{\vartheta } :\vartheta \in \varTheta \}$. In general, the space of parameters $\varTheta$ is endowed with a fixed $\sigma$-algebra $\mathcal {G}$. It is assumed that all singletons are in the $\sigma$-algebra, $\{ \vartheta \} \in \mathcal {G}$. Let us call $\mathbb {R}_+ = [0, \infty )$ the *time set*, $(X, \varrho )$ the *phase space*, and $(\varTheta , \mathcal {G})$ the *control set*. In this paper we shall operate mainly with finite subsets of a control set i.e. $\varTheta^{*} = \{ 0 , 1 , \dots , M \}\subseteq \varTheta$ (where for instance $\varTheta \ = \ [0,M]$). We have $\mathcal{G}^{*}= \mathcal {G}\cap \varTheta^{*} = 2^{\varTheta *}$ if $\varTheta^{*}$ finite.

### Definition 4

Take, as mentioned above, $\varTheta^{*} = \{ 0, 1, \ldots , M \}$ to be a finite subset of $\varTheta$. A function $\mathfrak {h} :[0, \infty ) \mapsto \varTheta^{*}$ is said to be piecewise constant if there exists a sequence $0 = t_{-1}< t_0< t_1< t_2< \cdots < t_n \rightarrow \infty$ such that $\mathfrak {h}(s) = \vartheta _n \in \varTheta^{*}$ for all $s\in [t_n, t_{n+1})$ with the additional restriction that $\vartheta _{n} \ne \vartheta _{n+1}$ for all *n*. The family of all piecewise constant functions (the sequences of break points $t_0< t_1< t_2< \cdots < t_n \rightarrow \infty$ may vary with $\mathfrak {h}$) will be denoted by $\mathfrak {H}(\varTheta^{*} )$. For a fixed function $\mathfrak {h} \in \mathfrak {H}(\varTheta^{*})$, the family of transformations (where $t\ge 0$)

$$\begin{aligned} \varPhi _t^{\mathfrak {h}} (x) = \varPhi ^{\vartheta _n}_{t-t_{n-1}} \circ \varPhi ^{\vartheta _{n-1}}_{t_{n-1} - t_{n-2}} \circ \cdots \circ \, \varPhi _{t_1-t_0}^{\vartheta _1}\circ \, \varPhi _{t_0}^{\vartheta _0}(x), \ \ \ \text {for } \ \ \ t\in [t_{n-1}, t_n ) , \end{aligned}$$

is called a *deterministically controlled* (by $\mathfrak {h}$) semiflow. This could be considered to be a deterministic version of a renewal process. The class of all deterministically controlled by $\mathfrak {h}$ semiflows (i.e. transformations $\varPhi _t^{\mathfrak {h}}(\cdot ), \ t\ge 0$) is denoted by $\mathfrak {R}^{\mathfrak {h}}(\varTheta^{*})$. We also define $\mathfrak {R}(\mathfrak {H}(\varTheta^{*})) = \bigcup _{\mathfrak {h}\in \mathfrak {H}(\varTheta^{*})} \mathfrak {R}^{\mathfrak {h}}(\varTheta^{*})$.

We now turn to discussing notations and other theoretical aspects. The proofs of the presented here lemmata and theorems can be found in the “Appendix”.

### Lemma 1

Endowed with the distance function

$$\begin{aligned} d_r(\mathfrak {h}, \mathfrak {g}) = \int _0^r \delta _{\mathfrak {h}(t), \mathfrak {g}(t)} \mathrm{d}t \ , \end{aligned}$$

the space $\mathfrak {H}_r(\varTheta^{*}) = \{ \mathfrak {h}\upharpoonright _{[0,r]} :\mathfrak {h} \in \mathfrak {H}(\varTheta^{*}) \}$ (where $$ \delta _{\mathfrak {h}(t),\mathfrak {g}(t) } = {\left\{ \begin{array}{ll} 1 , \ \text {if} \ \mathfrak {h}(t) \ne \mathfrak {g}(t), \\ 0 , \ \text {if} \ \mathfrak {h}(t) = \mathfrak {g}(t) \end{array}\right. }$$ is the Dirac delta operation) becomes a metric space.

### Remark 1

We skip the proof as it is obvious. Notice that the metric $d_r$ is equivalent to the $L^1$-norm distance $\int _0^r | \mathfrak {h}(t) - \mathfrak {g}(t) | \mathrm{d}t$. As the ranges of the functions from $\mathfrak {H}_r(\varTheta^{*})$ are confined to $\{ 0 , 1 ,\ldots , M \}$ thus (on $L^1([0,r])$)

$$\begin{aligned} \frac{1}{M} \Vert \mathfrak {h} - \mathfrak {g} \Vert _1 \le d_r( \mathfrak {h}, \mathfrak {g}) \le \Vert \mathfrak {h} - \mathfrak {g} \Vert _1 \ . \end{aligned}$$

In particular, $(\mathfrak {H}_r(\varTheta^{*}), d_r )$ is a separable metric space (not complete in general). We also notice that $d_r (\mathfrak {h}, \mathfrak {g}) = \lambda (\{ t\in [0,r] :\mathfrak {h}(t) \ne \mathfrak {g}(t)\})$.

### Lemma 2

Let $$\{ \varPhi ^{\vartheta }_t \}_{t\ge 0, \vartheta \in \varTheta } $$ be the family of semiflows on $$(X, \varrho )$$ such that for finite $$\varTheta^{*} \subseteq \varTheta $$ the conditions ([Disp-formula Equ2]) and ([Disp-formula Equ1]) hold, with a constant *L* and the growth function $$\varDelta $$ which is independent of $$\vartheta \in \varTheta^{*}$$. Given $$\mathfrak {h}, \mathfrak {g} \in \mathfrak {H}(\varTheta^{*})$$ such that $$\mathfrak {h}(0) = \mathfrak {g}(0)$$ , let $$w_{-1} = 0< w_0< w_1 < \cdots $$ be the sequence of real numbers, where the functions $$\mathfrak {h}, \mathfrak {g}$$ stop being equal or alternatively start being equal (i.e. $$w_0 = \min \{ t > 0 :\mathfrak {h}(t) \ne \mathfrak {g}(t) \},$$$$w_1 = \min \{ t > w_0 :\mathfrak {h}(t) = \mathfrak {g}(t) \},$$$$w_2 = \min \{ t > w_1 : \mathfrak {h}(t) \ne \mathfrak {g}(t) \}, \dots $$). For a fixed time parameter $$r > 0$$ let $$n_* \in \mathbb {N}$$ be such that $$w_{n_*} \le r < w_{n_* + 1}$$. Then for all $$x, y \in X$$ we can bound

$$\begin{aligned} \begin{aligned}&\varrho (\varPhi ^{\mathfrak {h}}_{r}(x) , \varPhi ^{\mathfrak {g}}_{r}(y)) \\&\quad \le 2L ( (r - w_{2j}) + (w_{2j-1} - w_{2j-2})\varDelta (w_{2j } - w_{2j-1}) \\&\qquad + (w_{2j-3} - w_{2j-4})\varDelta (w_{2j} - w_{2j-1})\varDelta (w_{2j-2} - w_{2j-3}) + \cdots \\&\qquad + (w_1 - w_0)\varDelta (w_{2j } - w_{2j-1}) + (w_{2j-3} - w_{2j-4})\varDelta (w_{2j} - w_{2j-1})\cdots \varDelta (w_2 - w_1) ) \\&\qquad + \varDelta (w_{2j } - w_{2j-1})\varDelta (w_{2j-2} - w_{2j-3})\cdots \varDelta (w_{2} - w_{1})\varDelta (w_0\wedge r - 0)\varrho (x,y) \ \end{aligned} \end{aligned}$$

if $n_* = 2j$ (the trivial case $r \le w_0$ will be discussed separately) and

$$\begin{aligned} \begin{aligned}&\varrho (\varPhi ^{\mathfrak {h}}_r (x) , \varPhi ^{\mathfrak {g}}_r(y)) \\&\quad \le 2L ((w_{2j-1} - w_{2j-2})\varDelta (r - w_{2j-1}) \\&\qquad + (w_{2j-3} - w_{2j-4})\varDelta (r - w_{2j-1}) \varDelta (w_{2j-2} - w_{2j-3}) \\&\qquad + (w_{2j-5} - w_{2j-6})\varDelta (r - w_{2j-1}) \varDelta (w_{2j-2} - w_{2j-3} \varDelta (w_{2j-4} - w_{2j-5})) + \cdots \\&\qquad + (w_1 - w_0)\varDelta (r - w_{2j-1}) \varDelta (w_{2j-2} - w_{2j-3} \cdots \varDelta (w_{2} - w_{1})) ) \\&\qquad + \varDelta (r - w_{2j-1}) \varDelta (w_{2j-2} - w_{2j-3} \cdots \varDelta (w_{2} - w_{1})\varDelta (w_0\wedge r - 0)\varrho (x,y) \ \end{aligned} \end{aligned}$$

if $$n_* = 2j-1$$.

Before the reader turns to the proof in the “Appendix” we notice that if $w_0 \ge r$, then evaluating $\varrho ( \varPhi ^{\mathfrak {h}}_r(x), \varPhi ^{\mathfrak {g}}_r(y) )$ and using the bounds from Lemma 2 we will simply obtain $\varrho ( \varPhi ^{\mathfrak {h}}_r(x), \varPhi ^{\mathfrak {g}}_r(y) ) \le \varDelta (r) \varrho (x, y)$; we remember that $\varDelta (0) =1$.

The next lemma is concerned with the case when $\mathfrak {h}(0) \ne \mathfrak {g}(0)$ and its proof is along the same lines as Lemma 2’s. For the completeness of the paper we include almost all the details.

### Lemma 3

Let $$\{ \varPhi ^{\vartheta }_t \}_{t\ge 0, \vartheta \in \varTheta } $$ be the family of semiflows on $$(X, \varrho )$$ such that, for some finite $$\varTheta^{*} \subseteq \varTheta $$, the conditions ([Disp-formula Equ2]) and ([Disp-formula Equ1]) hold, with a constant *L* and the growth function $$\varDelta $$ which is independent of $$\vartheta \in \varTheta^{*}$$. Given $$\mathfrak {h}, \mathfrak {g} \in \mathfrak {H}(\varTheta^{*})$$ such that $$\mathfrak {h}(0) \ne \mathfrak {g}(0)$$, let $$0 = w_0< w_1 < \cdots $$ be the sequence of real numbers, where the functions $$\mathfrak {h}, \mathfrak {g}$$ stop being equal or alternatively start being equal (i.e. $$w_1 = \min \{ t > 0 :\mathfrak {h}(t) = \mathfrak {g}(t) \},$$$$w_2 = \min \{ t > w_0 :\mathfrak {h}(t) \ne \mathfrak {g}(t) \},$$$$w_3 = \min \{ t > w_1 :\mathfrak {h}(t) = \mathfrak {g}(t) \}, \dots $$). For a fixed time parameter $$r > 0$$ let $$n_* \in \mathbb {N}$$ be such that $$w_{n_*} \le r < w_{n_* + 1}$$. Then for all $$x, y \in X$$ we have the bounds

$$\begin{aligned} \begin{aligned}&\varrho (\varPhi ^{\mathfrak {h}}_r(x) ,\varPhi ^{\mathfrak {g}}_r(y) ) \\&\quad \le 2L( (r - w_{2j}) + (w_{2j-1} - w_{2j-2})\varDelta (w_{2j} - w_{2j-1}) \\&\qquad + (w_{2j-3} - w_{2j-4} )\varDelta (w_{2j} - w_{2j-1})\varDelta (w_{2j-2} - w_{2j-3}) + \cdots \\&\qquad + (w_3 - w_2)\varDelta (w_{2j} - w_{2j-1})\varDelta (w_{2j-2} - w_{2j-3})\cdots \varDelta (w_4 - w_3) \\&\qquad + (w_1 - 0))\varDelta (w_{2j} - w_{2j-1})\varDelta (w_{2j-2} - w_{2j-3})\cdots \varDelta (w_4 - w_3)\varDelta (w_2 - w_1) \\&\qquad + \varDelta (w_{2j} - w_{2j-1})\varDelta (w_{2j-2} - w_{2j-3})\cdots \varDelta (w_4 - w_3)\varDelta (w_2 - w_1)\varrho (x,y) \ \end{aligned} \end{aligned}$$

if $$n_* = 2j$$ and

$$\begin{aligned} \begin{aligned}&\varrho (\varPhi ^{\mathfrak {h}}_r(x) ,\varPhi ^{\mathfrak {g}}_r(y) ) \\&\quad \le 2L( (w_{2j-1} - w_{2j-2})\varDelta (r - w_{2j-1}) \\&\qquad + (w_{2j-3} - w_{2j-4})\varDelta (r - w_{2j-1})\varDelta (w_{2j-2} - w_{2j-3}) + \cdots \\&\qquad + (w_3 - w_2)\varDelta (r - w_{2j-1})\varDelta (w_{2j-2} - w_{2j-3})\cdots \varDelta (w_4 - w_3) \\&\qquad + (w_1 - 0)\varDelta (r - w_{2j-1})\varDelta (w_{2j-2} - w_{2j-3})\cdots \varDelta (w_4 - w_3)\varDelta (w_2 - w_1) ) \\&\qquad + \varDelta (r - w_{2j-1})\varDelta (w_{2j-2} - w_{2j-3})\cdots \varDelta (w_4 - w_3)\varDelta (w_2 - w_1)\varrho (x,y) \end{aligned} \end{aligned}$$

if $$n_* = 2j-1$$.

We mention that $\varrho (\varPhi ^{\mathfrak {h}}_r(x) ,\varPhi ^{\mathfrak {g}}_r(y) ) \le 2Lr + \varrho (x,y)$ if $w_1 \ge r$.

Replacing the condition ([Disp-formula Equ2]) by the stronger one ([Disp-formula Equ3]), we obtain directly from the above lemmata:

### Corollary 1

Let $\{ \varPhi ^{\vartheta }_t \}_{t\ge 0, \vartheta \in \varTheta }$ be the family of semiflows on $(X, \varrho )$ such that, for some finite $\varTheta^{*} \subseteq \varTheta$, the conditions ([Disp-formula Equ3]) and ([Disp-formula Equ1]) hold, with a constant *L* and the growth function $\varDelta$ which is independent of $\vartheta \in \varTheta^{*}$. Given $\mathfrak {h}, \mathfrak {g} \in \mathfrak {H}(\varTheta^{*})$ and a fixed time parameter $r > 0$ we have the bounds

$$\begin{aligned} \varrho (\varPhi ^{\mathfrak {h}}_{r}(x) , \varPhi ^{\mathfrak {g}}_{r}(y)) \le 2LC \varDelta (r) d_r(\mathfrak {h}, \mathfrak {g}) + C\varDelta (r)\varrho (x,y) \ . \end{aligned}$$

If $$x=y$$, then

$$\begin{aligned} \varrho (\varPhi ^{\mathfrak {h}}_{r}(x) , \varPhi ^{\mathfrak {g}}_{r}(x)) \le 2LC \varDelta (r) d_r(\mathfrak {h}, \mathfrak {g}) \ . \end{aligned}$$

In particular, the mapping $\mathfrak {H}_r(\varTheta^{*}) \ni \mathfrak {h} \mapsto \varPhi ^{\mathfrak {h}}_r(x) \in X$ is $(d_r, \varrho )$ continuous.

By $\mathfrak {H}_{r;m}(\varTheta^{*})$ we denote the set of all $\mathfrak {h}\in \mathfrak {H}_r(\varTheta^{*})$ such that $\mathfrak {h}$ changes its values at most *m* times on the interval [0, *r*]. We obtain the next corollary.

### Corollary 2

Let $\{ \varPhi ^{\vartheta }_t \}_{t\ge 0, \vartheta \in \varTheta }$ be the family of semiflows on $(X, \varrho )$ such that, for some finite $\varTheta^{*} \subseteq \varTheta$, the conditions ([Disp-formula Equ2]) and ([Disp-formula Equ1]) hold, with a constant *L* and the growth function $\varDelta$ which is independent of $\vartheta \in \varTheta^{*}$. Given $\mathfrak {h}, \mathfrak {g} \in \mathfrak {H}_{r;m}(\varTheta^{*})$ and arbitrary $x, y \in X$ we have the bounds

$$\begin{aligned} \varrho (\varPhi _r^{\mathfrak {h}}(x) , \varPhi _r^{\mathfrak {g}}(y) ) \le 2 L \varDelta (r)^{2m} d_r(\mathfrak {h}, \mathfrak {g}) + \varDelta (r)^{2m}\varrho (x,y) \end{aligned}$$

and

$$\begin{aligned} \varrho (\varPhi _r^{\mathfrak {h}}(x) , \varPhi _r^{\mathfrak {g}}(x) ) \le 2 L \varDelta (r)^{2m} d_r(\mathfrak {h}, \mathfrak {g}) . \end{aligned}$$

The following theorem will be used in what will follow. It is a straightforward consequence of the already proved lemmata and corollaries.

### Theorem 1

Let $\{ \varPhi ^{\vartheta }_t \}_{t\ge 0, \vartheta \in \varTheta }$ be the family of semiflows on $(X, \varrho )$ such that, for some finite $\varTheta^{*} \subseteq \varTheta$, the conditions ([Disp-formula Equ2]) and ([Disp-formula Equ1]) hold, with a constant *L* and the growth function $\varDelta$ which is independent of $\vartheta \in \varTheta^{*}$. Then, for every fixed $x\in X$ the mapping

$$\begin{aligned} \mathfrak {H}_{r;m}(\varTheta^{*}) \ni \mathfrak {h} \mapsto \varPhi ^{\mathfrak {h}}_r(x) \in X \end{aligned}$$

is $$(d_r , \varrho )$$ continuous.

Now let us relax $\mathfrak {h}$ and allow it to be the trajectories of a fixed continuous time homogeneous Markov chain (CTHMC) on $\varTheta^{*}$. It is well known (cf. Allen 2011, p. 200; Norris 2007 p. 73, 94), that in this case we have $\xi _\circ (\omega ) \in \mathfrak {H}(\varTheta^{*})$ for almost all $\omega \in \varOmega$. Of course, the sequences of switching times $t_0(\omega )< t_1(\omega )< t_2(\omega ) < \ldots$ are not fixed anymore (they do depend on a particular $\omega \in \varOmega$), and therefore our control functions $\mathfrak {h}^{\omega }(\cdot ) = \xi _{\circ }(\omega )$ may vary frequently. We describe this notion in all details by introducing first:

### Definition 5

Let $(\varOmega , \mathcal {F}, P)$ be a (complete) probability space. A stochastic process $\{ \xi _t \}_{t\in [0,\infty )}$ is called a control process if $\xi _t :\varOmega \mapsto \varTheta^{*}$.

A Markovian control process is a control process $\{ \xi _t \}_{t\in [0, \infty )}$, which is a continuous time homogeneous Markov process on a measurable space $(\varTheta^{*} , \mathcal {G})$. We denote

$$\begin{aligned} P_{\vartheta } (\xi _t \in \cdot ) = P(\xi _t \in \cdot \ \ | \ \xi _0 = \vartheta ) \ . \end{aligned}$$

Our definitions and notations concerning Markov processes are standard and are borrowed from Allen (2011), Gikhman and Skorokhod (1974), Grimmett and Stirzaker (2009), Norris (2007). Abbreviating, we shall denote the process $\{ \xi _t \}_{t\in [0, \infty )}$ by $\varXi$. In the case, when the control phase space $\varTheta^{*}$ is finite, continuous time homogeneous Markov chains (CTHMC) are very simple and intuitive stochastic objects. Their dynamics and random evolution is fully governed by the so-called *Q* matrices Norris (2007). A *Q* matrix is a real square matrix

$$\begin{aligned} Q = \left[ \begin{array}{ccccc} -q_{0,0} &{} q_{0,1} &{} q_{0,2} &{}\cdots &{} q_{0,M} \\ q_{1,0} &{} -q_{1,1} &{} q_{1,2} &{} \cdots &{} q_{1, M} \\ \vdots &{} \vdots &{} \vdots &{} \vdots &{} \vdots \\ q_{M,0} &{} q_{M,1} &{} q_{M,2} &{} \cdots &{} - q_{M,M} \end{array} \right] _{(M+1)\times (M+1)} \ . \end{aligned}$$

such that all $q_{j,k} \ge 0$, and for every $j\in \varTheta^{*}$ we have $\sum _{l=0, l\ne j}^M q_{j,l} = q_{j,j}$. It follows that in the matrix *Q* the sum of elements in every row is 0. We will write $q_j$ instead of $q_{j,j}$. The matrix *Q* is called the *intensity matrix* of the process $\varXi$ as long as for every $j \in \varTheta^{*}$ we have

$$\begin{aligned} \lim _{h\rightarrow 0^+} \frac{P(\xi _{t+h} \ne j | \xi _t = j )}{h} = q_j \ \text {and} \ \ \lim _{h\rightarrow 0^+} \frac{P(\xi _{t+h} = k | \xi _t = j )}{h} = q_{j,k} \ \text {for} \ k \ne j \ . \end{aligned}$$

Formally, we may define the matrix *Q* as

$$\begin{aligned} Q_{k,j} = \lim _{h \rightarrow 0^+} \frac{p_{k,j}(h) - \delta _{k,j}}{h} \ \ . \end{aligned}$$

We skip other formalisms and direct the reader to classical monographs (cf. Gikhman and Skorokhod 1974), where all the essential results with clear proofs are presented. The intensity matrices (generators) are at the core of the analytical theory of Markov semigroups.

The trajectory of a process $\varXi$ may be simulated using its *Q* matrix, by the classical Gillespie algorithm (Gillespie 1977). Namely, a trajectory starting at $t_\bullet = 0$, from an initial point $\vartheta _0 \in \varTheta^{*}$, will stay in $\vartheta _0$ for a random time $\tau _{\vartheta _0}$. Then, at the moment $t_1 = \tau _{\vartheta _0}$, it jumps with probability $\dfrac{q_{\vartheta _0, k}}{q_{\vartheta _0}}$ to some $k \ne \vartheta _0$. The scheme is repeated infinitely many times. As the process is Markovian, the distribution of $\tau _\vartheta$, where $\vartheta \in \varTheta^{*}$ is exponential; i.e. $P(\tau _\vartheta > t) = \mathrm{e}^{- q_{\vartheta }t}$, for $t \ge 0$ (see Norris 2007, p. 94). From the point of view of simulations, the full description of the trajectory (for a fixed $\omega \in \varOmega$) is a sequence of pairs

$$\begin{aligned} \left( (\vartheta _0, t_0), (\vartheta _1 , t_1 ), (\vartheta _2 , t_2), \ldots \right) \ , \end{aligned}$$

where $\vartheta _n$ describe consecutive states of the trajectory of $\varXi$. The sequence of times $t_0< t_1< t_2 < \cdots$ represents instances of switching. Then, $t_0 = \tau _{\vartheta _0 }$, $t_1 = t_0 + \tau _{\vartheta _1} = \tau _{\vartheta _0} + \tau _{\vartheta _1}$, $t_2 = t_1 + \tau _{\vartheta _2 }$ and so forth. We must notice that our presentation is a bit informal, as we skip over the technicality that all random variables (Markov moments) $\tau _{\vartheta _0}, \tau _{\vartheta _1}, \tau _{\vartheta _2}, \ldots$ are actually versions from the relevant families of independent copies. Obviously, $t_n(\omega )$ is the time instance of the jump $\vartheta _n \rightsquigarrow \vartheta _{n+1}$.

Given a CTHMC $\varXi$ we shall denote by $\{ \eta _n \}_{n\ge 0}$ the embedded Markov chain and the sequence of the so-called inter-times of $\varXi$ by $\{ \iota _n \}_{n\ge 0}$. Of course $\eta _n$ is a discrete time Markov chain on the control phase space $\varTheta^{*}$ and $\iota _n$ form a sequence of independent and nonnegative random variables, which describe the (waiting) time spent in $\eta _n(\omega )$ before jumping to $\eta _{n+1}(\omega )$. To keep formulæ short (this repetition is consistent with the one introduced a while ago), let us denote $t_n(\omega ) = \iota _0(\omega ) + \cdots +\iota _{n}(\omega )$, it is the time when the process $\xi _t(\omega )$ (after occupying parameter $\eta _n(\omega )$ for the period $\iota _n(\omega )$) arrives at the state $\eta _{n+1}(\omega )$ (being then “stuck” in it for the period $\iota _{n+1}(\omega )$).

### Definition 6

Let $\varXi$ be a CTHMC defined on the probability space $(\varOmega , \mathcal {F}, P)$ with values in a finite control phase space $(\varTheta^{*} , \mathcal {G})$ and, as before, let $\mathfrak {R}(\varTheta^{*}) \ = \ \{ \varPhi (t, \cdot , \vartheta ) :t\ge 0 , \vartheta \in \varTheta^{*} \ \}$ be a family of semiflows on the phase space $(X, \varrho )$. Then the mapping

$$\begin{aligned} \zeta :[0, \infty ) \times X \times \varOmega \mapsto X \end{aligned}$$

defined as $$\zeta (t, x, \omega ) = \varPhi ^{\xi _{\circ }(\omega )}_t(x)$$ i.e.

$$\begin{aligned} \begin{aligned}&\zeta (t,x,\omega ) & = \varPhi ^{\xi _{\circ }(\omega )}_t(x)\\&\quad = \varPhi (t, x, \vartheta ) \ \ \ \ \text {for} \ \ 0 \le t \le \iota _0(\omega ) = t_1(\omega ) \ \text {and} \ \vartheta =\eta _0(\omega ) , \\&\quad = \varPhi (t - t_1(\omega ), \zeta (t_1(\omega ), x , \eta _0(\omega ) ), \eta _1(\omega )) \ \text {for} \ \ t_1(\omega ) \le t \le t_2(\omega ), \\&\quad = \varPhi (t - t_2(\omega ), \zeta (t_2(\omega ), x , \omega )) , \eta _2(\omega )) \ \ \ \text {for} \ \ t_2(\omega ) \le t \le t_3(\omega ), \\&\qquad \qquad \text {and then inductively } \ \ \\&\quad = \varPhi (t - t_n(\omega ) , \zeta (t_n(\omega ), x , \omega ) , \eta _n(\omega ) ) \ \ \ \text {for} \ \ \ t_n(\omega ) \le t \le t_{n+1}(\omega ), \ \end{aligned} \end{aligned}$$

is called a *randomly controlled semiflow* (which is a merged system of the family of the semiflows $\mathfrak {R}(\varTheta^{*})$ and the control CTHMC process $\varXi = \{ \xi _t \}_{t\in [0,\infty )}$ on the control phase space $\varTheta^{*}$). We denote such a randomly controlled family of semiflows by $\mathfrak {R}(\varTheta^{*} ) \divideontimes \varXi$.

Each $\zeta \in \mathfrak {R}(\varTheta^{*} ) \divideontimes \varXi$ is in fact a random process. For $t \in \mathbb {R}_+$ we introduce

$$\begin{aligned} \xi ^{\diamond + t}_r(\omega ) = \xi _{r + t}(\omega ) \ \text {for \ all} \ r\ge 0. \end{aligned}$$

Of course $\xi ^{\diamond + t}_{\circ }(\omega ) \in \mathfrak {H}(\varTheta^{*})$ for *P*-almost all $\omega \in \varOmega$. It follows from our construction that $\zeta (0, x, \cdot ) = x$. Moreover, if we denote $\zeta _t(x, \omega ) = \zeta (t,x,\omega )$ then

### Theorem 2

Let $\{ \varPhi ^{\vartheta }_t \}_{t\ge 0, \vartheta \in \varTheta }$ be the family of semiflows on $(X, \varrho )$ such that, for finite $\varTheta^{*} \subseteq \varTheta$, the conditions ([Disp-formula Equ2]) and ([Disp-formula Equ1]) hold, with a constant *L* and the growth function $\varDelta$ which is independent of $\vartheta \in \varTheta^{*}$. If $\varXi = \{ \xi _t \}_{t\in [0,\infty )}$ is the control CTHMC process on $\varTheta^{*}$, then $\{ \zeta _t (x, \cdot ) :t\in [0, \infty ) \}$ is a continuous time stochastic process on *X* with continuous trajectories.

## Simulation example: a simple linear random dynamical system

We consider two simulation examples to illustrate the behaviour of the linear random dynamical system $\varPhi ^{a}_{t}(x)=x+at$. The first one is very simple and plays here an illustrative role. The second one is related to recently studied SARS-CoV-2 dynamics. One of the purposes of the simulation is to illustrate, similarly as will later be observed for SIR models, the limiting, as the frequency of change in the driving process increases, behaviour of the considered $\varPhi ^{a}$ semiflow. Let *a* be a Markov process on a discrete state space $\{0,\ldots ,M\}$ with transition rate matrix *Q*. Assume that *a* starts from 0. Let $\mu$ be the stationary measure of the Markov chain *a*. Define $a^{*}= \lim \limits _{k\rightarrow \infty } (\epsilon _{0}+\cdots +\epsilon _{k})/(k+1)$, where $\{\epsilon _{0},\ldots ,\epsilon _{k}\}$ are i.i.d as $\mu$. The hypothesis is then, that $\varPhi ^{a^{(k)}}_{t}\rightarrow \varPhi ^{a^{*}}_{t}$ as $k\rightarrow \infty$, where $a^{(k)}$ is a Markov process with transition rate matrix *kQ*, i.e. the matrix *Q* multiplied by the integer *k*. We assume that the parameter *a* is the realization of a Markov process on the state space $\{0,1,2\}$ with rate matrix

$$\begin{aligned} Q_{1}= \left[ \begin{array}{ccc} -1 &{} 1 &{} 0 \\ 1 &{} -2 &{} 1 \\ 0 &{} 3 &{} -3 \end{array} \right] . \end{aligned}$$

**Fig. 1:**
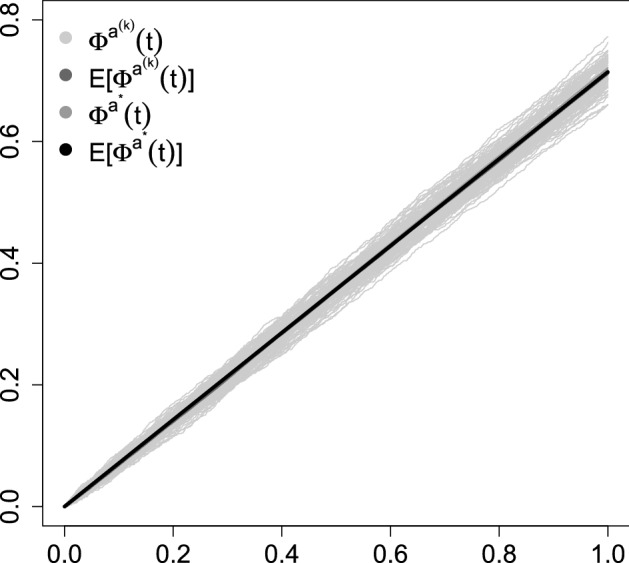
Simulated trajectories of $\varPhi ^{a^{(k)}}_{t}$ and $\varPhi ^{a^{*}}_{t}$ for $Q_{1}$. The curves ${\text {E}}\left[ \varPhi ^{a^{(k)}}_{t} \right]$, $\varPhi ^{a^{*}}_{t}$ and ${\text {E}}\left[ \varPhi ^{a^{*}}_{t} \right]$ essentially coincide with each other

We take this *Q* matrix for illustrative purposes, not due to some particular application. We first simulate the trajectory of $a^{(k)}$ for a large value of $n=100{,}000$. Then conditional on it, we calculate $\varPhi ^{a}_{t}$. Simulation of the Markov process is done by the Gillespie algorithm. We find $a^{*}$ by simulations. First from $Q_{1}$ we find the stationary distribution $\mu _*$ as the first row of the matrix $\mathbf {P}e^{10{,}000 \varvec{\Lambda }}\mathbf {P}^{-1}$, where $\varvec{\Lambda }$ is the diagonal matrix of eigenvalues of *Q* and $\mathbf {P}$ is the matrix of eigenvectors (Thm. 21, Grimmett and Stirzaker 2009). The 10, 000 is the to represent $\infty$, i.e. the limit of $e^{tQ_{1}}$ as $t\rightarrow \infty$. From $\mu _*$ we draw a large (10, 000) sample and take its average as $a^{*}$. We simulate both $a^{(k)}$ and $a^{*}$ 100 times. We show the resulting collection of trajectories in Fig. 1.

Next we aim at providing an example linked to the dynamics of the SARS-CoV-2 virus. Let the process $\varPhi ^{a}_{t}$ represent a short term approximation of the growth of the number of infected, i.e. at a stage of linear growth. The infected number, as described in the Introduction, is driven by a random factor. In the common SIR model (as will be described in Sect. 4) the number of infected is given by the differential equation

$$\begin{aligned} I'(t) = (\beta S(t) - \alpha )I(t) \end{aligned}$$

and conditional on $S(t) \equiv S$ we have the (conditional) solution

$$\begin{aligned} I(t) = e^{(\beta S-\alpha )t} \approx I(0) + (\beta S -\alpha ) t = I(0) + \alpha (\frac{\beta }{\alpha } S -1) t, \end{aligned}$$

where we can take $\frac{\beta }{\alpha } S = R_{0}$. Hence, to compare with our model we would like $a(t) = \alpha (\frac{\beta }{\alpha } S -1)$, where some part of the right-hand side is time-dependent. Alternatively, we would like $a^{*} = \alpha (\frac{\beta }{\alpha } S -1)$. To connect this with the dynamics of the SARS-CoV-2 virus one would need estimates of $\alpha$, $R_{0}$ for it and some population size *S*(*t*). Obtaining $\alpha$ and $R_{0}$ from official statistics does not seem to be an easy task due to the nature of the collected data (Bartoszek et al. 2020). In particular the number of confirmed cases strongly depends on the number of tests performed and furthermore one can suspect that the count will also depend on the testing strategy and type of test performed. However, one alternative is to use data collected from the cruise ship, *Diamond Princess*, which can be treated as a unique, naturally-occurring epidemiological study that could be useful for the prediction of parameters related to the behaviour of COVID-19. There were 3711 people onboard, 1045 crew (median age 36 years) and 2666 passengers (median age 69 year) (Moriarty et al. 2020). Of these 712 tested positive for the virus, 145 crew and 567 passengers^1^. The first case was observed at the end of January 2020 and by March 2020 all passengers and crew had disembarked (Moriarty et al. 2020). Fourteen passengers died, with the first death on $10{\mathrm {th}}$ February 2020 and last on $14{\mathrm {th}}$ April 2020 (see Footnote 1). We need to have estimates of $\alpha$, the death rate, and $R_{0}$. For the death rate we take (approximating the 64 days as 2 months),

$$\begin{aligned} \alpha = \frac{1}{\mathrm {time}}\cdot \frac{\mathrm {number of deaths}}{\mathrm {population size}} = \frac{1}{2\mathrm {months}}\cdot \frac{14}{567} \approx 0.012. \end{aligned}$$

There are various estimates of $R_{0}$ for the *Diamond Princess*, 14.8, 1.78 (Rocklöv et al. 2020) and 2.28 (Zhang et al. 2020). Here we take the value of 3.7 (the reported by Liu et al. 2020 mean $R_{0}$ value from Wuhan and it was taken in Rocklöv et al. (2020) for a sensitivity analysis). We hence obtain $a^{*}_{DP} = ((14/567)/2)\cdot 3.7 \approx 0.046$. Of course, in order to be able to carry out our simulation we need to have *Q*, of which $a^{*}$ is a function of. Let $\mathcal {Q}\subset \mathbb {R}^{3\times 3}$ be the space of matrices that are constrained to have non-negative off-diagonals, each row summing to 0 and at least one non-zero off-diagonal entry in each row. Each $Q\in \mathcal {Q}$ will then be the transition rate matrix for some Markov process and have the $a^{*}_{Q}$ parameter associated with. Our aim is to find a *Q* that minimizes

3.1 $$\begin{aligned} \tilde{Q} = \mathop {\hbox {argmin}}\limits _{Q \in \mathcal {Q}} (a^{*}_{Q}-a^{*}_{DP})^{2}. \end{aligned}$$

**Fig. 2:**
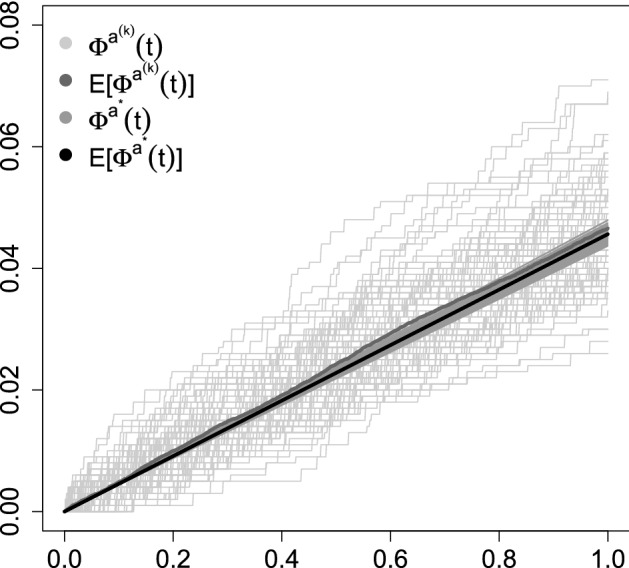
Simulated trajectories of $\varPhi ^{a^{(k)}}(t)$ and $\varPhi ^{a^{*}}(t)$ for *Q*s optimized in order to have $a^{*}$ agreeing with $a^{*}_{DP}$

One cannot expect to have a unique $\tilde{Q}$, as multiple *Q*s can result in the same $a^{*}$ and hence we want to consider a whole set of viable $\tilde{Q}$s. We therefore take a Monte Carlo approach by running R’s (R Core Team 2020) optim() function from 100 random seeds sampled from $\mathcal {M}$. A given $Q\in \mathcal {M}$ is randomly chosen as follows. First for each row the position of the obligatory positive-off-diagonal entry is chosen. Then, from the remaining $M-1$ (in this application only 1 entry remains, as $M=2$) entries each one is chosen with probability 0.5 (we first draw from the binomial distribution the number of positive entries, and then using R’s sample() draw the appropriate indices). The values of the chosen entries are drawn from the exponential with rate 1 distribution. Afterwords, the optimization of Eq. (3.1) is done using the Nelder–Mead method. We show the resulting collection of trajectories in Fig. 2.

## Worked example: the SIS model with random coefficients

We finally turn to the investigation of trajectories of SIS (this Section) and SIR (Sect. 5) models controlled by a CTHMC with intensity matrix *Q* (and an initial distribution *p*) on finite state space $\{0,1,\ldots ,M\}$ (the numbers $0,1,\ldots ,M$ here are just labels, the actual states of the process will be the values of the transmission rates).

The classical SIS model for a constant population size Allen (2011), Kermack and McKendrick (1927) is described by a system of ODEs with constant coefficients (we remind the reader that without loss of generality we have assumed $N=1$, so in the more general case $\beta$ should be scaled by *N* and $S(t)+I(t)=N$):

4.1 $$\begin{aligned} \begin{aligned} S'(t)&= -\beta \ S(t)\ I(t) + \alpha \ I(t) \\ I'(t)&=\beta \ S(t)\ I(t) - \alpha \ I(t), \end{aligned} \end{aligned}$$

$S(t)+I(t)=1$. The parameter $\beta$ is called the transmission rate—the number of contacts per time that results in an infection of a susceptible individual; $\alpha$ is the recovery rate, where $1/\alpha$ is the average length of the infectious period.

As we have normalized out variables ($N=1$) we may consider solely the number of infected (and infectious) individuals *I*(*t*) at time *t* (as the number of susceptible individuals is directly given as $S(t)=1-I(t)$):

$$\begin{aligned} \frac{\mathrm{d}I(t)}{\mathrm{d}t}=\beta (1-I(t))\ I(t)-\alpha \ I(t)=-\beta \ I^2(t) + (\beta -\alpha )\ I(t) \end{aligned}$$

The above Bernoulli differential equation can be solved directly; the solution *I*(*t*) is given by

$$\begin{aligned} I(t)=\frac{\beta -\alpha }{\beta +\left( \frac{\beta -\alpha }{I(0)}-\beta \right) e^{-(\beta -\alpha )t}},\ t\ge 0. \end{aligned}$$

The SIS epidemic model has an endemic equilibrium given by

$$\begin{aligned} S^{*}=\frac{\alpha }{\beta },\ I^{*}=\frac{\beta -\alpha }{\beta }, \end{aligned}$$

and it is positive if the basic reproduction number

$$\begin{aligned} R_0=\frac{\beta }{\alpha } \end{aligned}$$

satisfies $R_0>1$. In that case, the solutions approach the endemic equilibrium. If $R_0\le 1$ solutions approach the infected-free state.

The first SIS model is controlled by the CTHMC through the parameter $\beta$ (which, after further modifications, we denote by $\beta _{1}$) which equals the value of the CTHMC.

For our simulation let us assume that the transmission rate $\beta =\beta (t)$ is the realization of a CTHMC on the state space {0, 0.044, 0.11, 0.22} with intensity matrix

$$\begin{aligned} Q=\begin{bmatrix} -1 &{} 1 &{} 0 &{} 0 \\ 0 &{} -1 &{} 1 &{} 0 \\ 0 &{} 1 &{} -2 &{} 1 \\ 1 &{} 0 &{} 0 &{} -1 \end{bmatrix}. \end{aligned}$$

From *Q* we find the stationary distribution ($\mu _*\circ Q=0$): $\mu _*=(0.2,0.4,0.2,0.2)$. We note that the particular values of *Q* are chosen for illustrative purposes only.

**Fig. 3:**
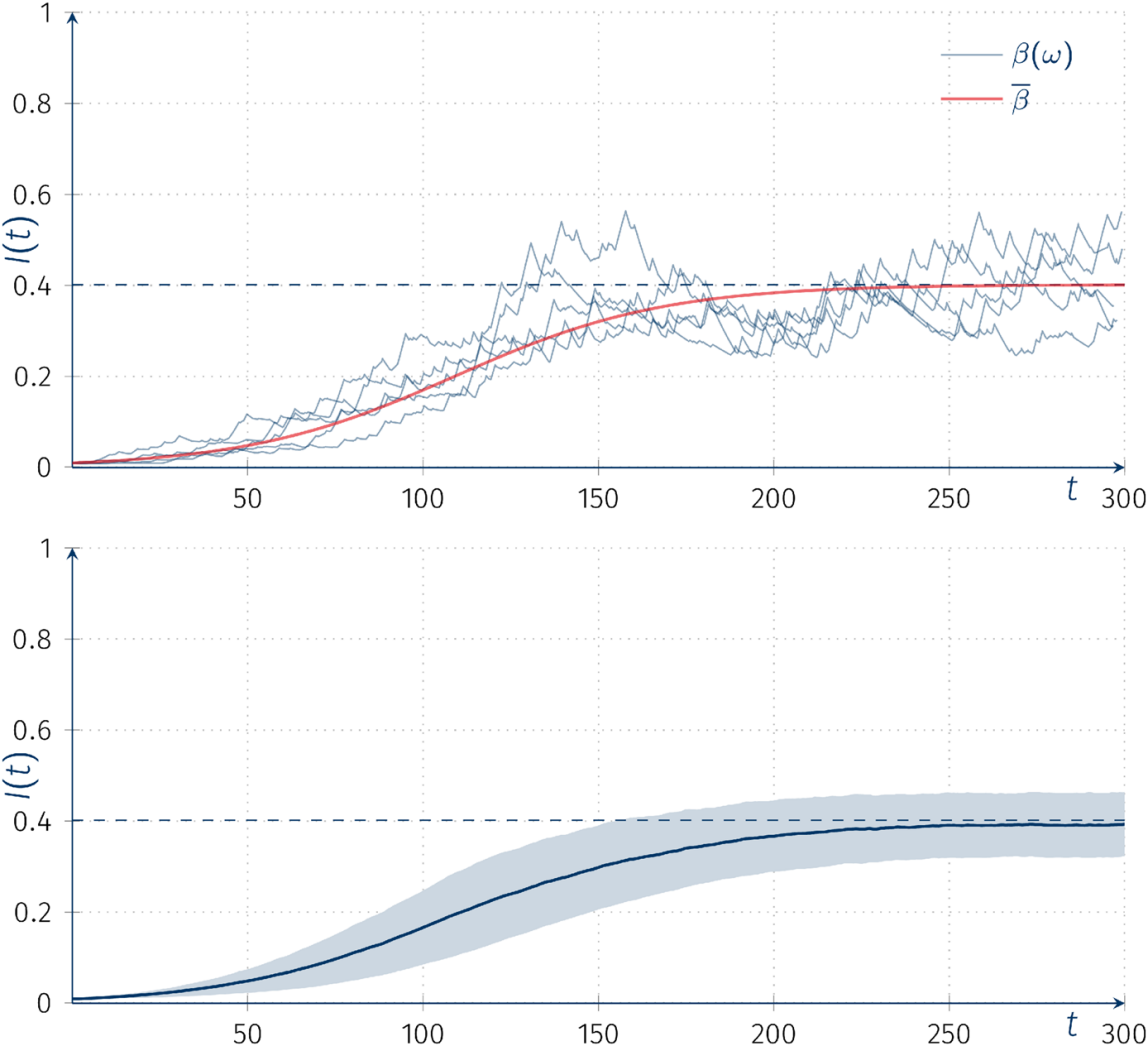
The SIS model with random coefficients. Top: simulated trajectories of $I(t)(\omega )$ from stochastic process $\beta (t)$ with *Q* and $P(\beta (0)=0)$ compared with deterministic solution of ODE, $\frac{\mathrm{d}I(t)}{\mathrm{d}t}$, with parameter $\beta =\overline{\beta }=0.0836$, other model parameters: $\alpha =0.05, I(0)=0.01$; bottom: mean function of $L=1000$ trajectories with ± sd intervals

Figure 3 shows the trajectories of the piecewise-deterministic Markov process $I(t)(\omega )$ (top panel), as well as the mean of $L=1000$ trajectories (bottom panel), superimposed on the ordinary deterministic solution *I*(*t*) with parameter $\overline{\beta }$ corresponding to $\overline{\beta }\equiv {\text {E}}\left[ \beta \right] =\mu _*\circ (0,0.044,0.11,0.22)$.

We postulate the limiting behavior of trajectories as we increase the intensities of jumps in CTHMC, i.e. the intensity matrix of the process, *kQ* changes as $k \rightarrow \infty$. Only numerical simulations were considered. However, our framework, considering limits of *kQ* as $k\rightarrow \infty$ is consistent with known theorems describing conditions under which a sequence of Markov processes will converge to the solution of a system of first order ODEs (Kurtz 1971).

**Fig. 4:**
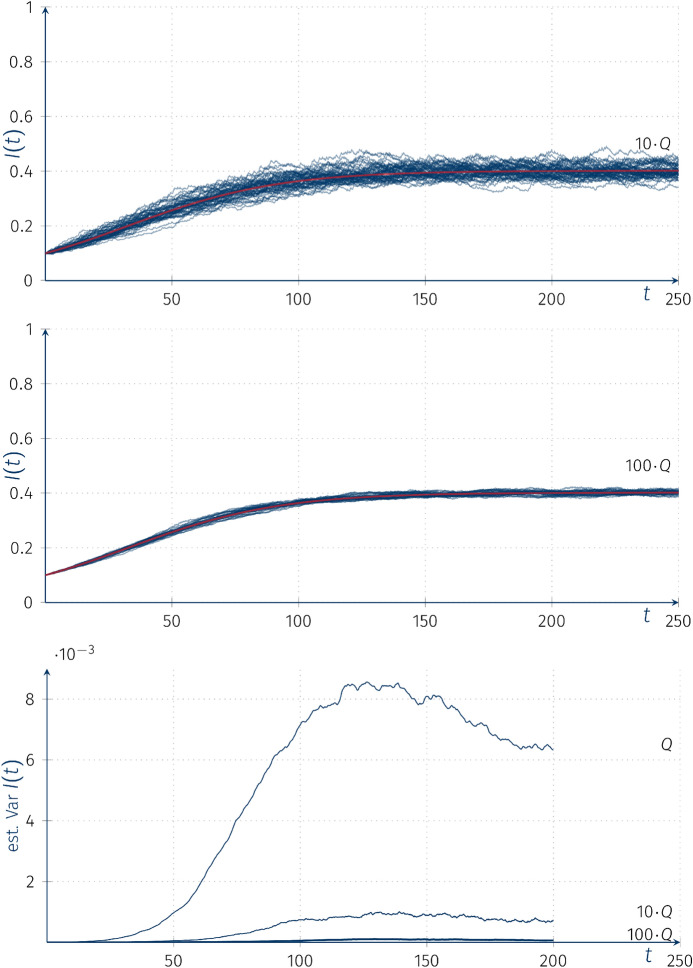
Trajectories of the piecewise-deterministic Markov processes $I(t)(\omega )$ for SIS models with random coefficients with intensity matrices 10*Q* and 100*Q*, and a comparison of the variance functions for models with intensity matrix *Q*, 10*Q* and 100*Q*

Figure 4 shows the trajectories of piecewise-deterministic Markov processes $I(t)(\omega )$ with intensity matrices 10*Q* and 100*Q* superimposed on ordinary deterministic solution *I*(*t*) with parameter $\overline{\beta }$ corresponding to $\mu _*\circ (0,0.044,0.11,0.22)$.

We now turn to considering a different stochastic modification of the SIS model. The spread of infection in the classical (deterministic) SIS model is associated with an incidence rate that is bilinear with respect to the number of susceptible and infected individuals. We propose a modification as we incorporate an “external” transmission of disease obtaining a non-multiplicative (affine) incidence rate (Korobeinikov and Maini 2005). Motivations for such an external transmission have been provided in the Introduction and were also discussed in Sect. 3.

As a consequence we obtain the following ODE system, again for a constant-sized population ($N=1$) SIS model:

$$\begin{aligned} \begin{aligned} \frac{\mathrm{d}S(t)}{\mathrm{d}t}&= -\beta _1 S(t)\ I(t) -\beta _0 S(t) + \alpha I(t) \\ \frac{\mathrm{d}I(t)}{\mathrm{d}t}&=\ \beta _1 S(t)\ I(t) +\beta _0 S(t) - \alpha I(t), \end{aligned} \end{aligned}$$

where for all $t>0$, $S(t)+I(t)=1\equiv \text {const.}$

From the above relation we expand the equation for the number of infected individuals *I*(*t*):

$$\begin{aligned} \frac{\mathrm{d}I(t)}{\mathrm{d}t}&=\ \beta _1 S(t)\ I(t) +\beta _0 S(t) - \alpha I(t)=\\&=\ \beta _1 (1-I(t))\ I(t) +\beta _0 (1-I(t)) - \alpha I(t)\\&= -\beta _1 \ I^2(t) +\left( \beta _{1}-\beta _0-\alpha \right) \ I(t) +\beta _{0}\\&= -\beta _1 \ I^2(t) +\gamma \ I(t) +\beta _{0}, \end{aligned}$$

where $$\gamma =\beta _{1}-\beta _0-\alpha $$.

We obtain again a first-order ODE, quadratic in the unknown function. However, our slight modification results in a significantly different “full-form” Riccati equation with constant coefficients (assuming $\beta _1, \beta _0>0$).

We now turn to solving our ODE. We first notice that a particular solution *i*(*t*) is:

$$\begin{aligned} 0&=-\beta _1 \ I^2(t) +\gamma \ I(t) +\beta _{0}\\ \end{aligned}$$

$$\gamma ^2+4\beta _0\beta _1>0$$

$$\begin{aligned} i_1(t)&=\frac{\gamma +\sqrt{\gamma ^2+4\beta _0\beta _1}}{2\beta _1}\\ i_2(t)&=\frac{\gamma -\sqrt{\gamma ^2+4\beta _0\beta _1}}{2\beta _1}\le 0, i(t)&\equiv \frac{\gamma +\sqrt{\gamma ^2+4\beta _0\beta _1}}{2\beta _1}>0. \end{aligned}$$

The “external” SIS epidemic model has an endemic equilibrium given by

$$\begin{aligned} S^{*}=\frac{\beta _1+\beta _0+\alpha -\sqrt{\gamma ^2+4\beta _0\beta _1}}{2\beta _1},\ I^{*} =\frac{\gamma +\sqrt{\gamma ^2+4\beta _0\beta _1}}{2\beta _1}, \end{aligned}$$

where $\gamma =\beta _1-\beta _0-\alpha$. Assuming $\beta _1, \beta _0>0$ the endemic equilibrium is always positive, i.e. there is no infected free state. Solving by quadrature, substituting $I(t)=i(t)+u(t)$, where $i'(t)=-\beta _1i^2(t)+\gamma i(t)+\beta _0$,

$$\begin{aligned} i'(t)+u'(t)&= -\beta _1 \ (i(t)+u(t))^2 +\gamma \ (i(t)+u(t)) +\beta _0,\\ u'(t)&= -\beta _1 u^2(t) +\gamma \ u(t) -2\beta _1i(t)\ u(t)\\&= -\beta _1 u^2(t) +(\gamma -2\beta _1i(t))\ u(t), \end{aligned}$$

or

$$\begin{aligned} u'(t)-(\gamma -2\beta _1i(t))\ u(t)=-\beta _1 u^2(t) \end{aligned}$$

which is a Bernoulli equation, (the coefficient $-(\gamma -2\beta _1i(t))$ equals$\sqrt{\gamma ^2+4\beta _0\beta _1}\ge 0$). Thus, substituting $I(t)=i(t)+\frac{1}{z(t)}$ directly into our Riccati equation yields (the linear equation):

$$\begin{aligned} z'(t)-\sqrt{\gamma ^2+4\beta _0\beta _1}\ z(t)=-\beta _1. \end{aligned}$$

A set of solutions to the Bernoulli equation is given by

$$\begin{aligned} u(t)=\frac{\sqrt{\gamma ^2+4\beta _0\beta _1}}{C_1 e^{\sqrt{\gamma ^2+4\beta _0\beta _1}\ t}-\beta _1}. \end{aligned}$$

A set of solutions to the Riccati equation is then given by

$$\begin{aligned} I(t)=\frac{\gamma +\sqrt{\gamma ^2+4\beta _0\beta _1}}{2\beta _1}+\dfrac{1}{C_2\ e^{\sqrt{\gamma ^2+4\beta _0\beta _1}\ t}-\frac{\beta _1}{\sqrt{\gamma ^2+4\beta _0\beta _1}}}. \end{aligned}$$

Assuming $$I(0)=I_0$$:

$$\begin{aligned} C_2=\left( I_0-\frac{\gamma +\sqrt{\gamma ^2+4\beta _0\beta _1}}{2\beta _1}\right) ^{-1}+\frac{\beta _1}{\sqrt{\gamma ^2+4\beta _0\beta _1}}. \end{aligned}$$

**Fig. 5:**
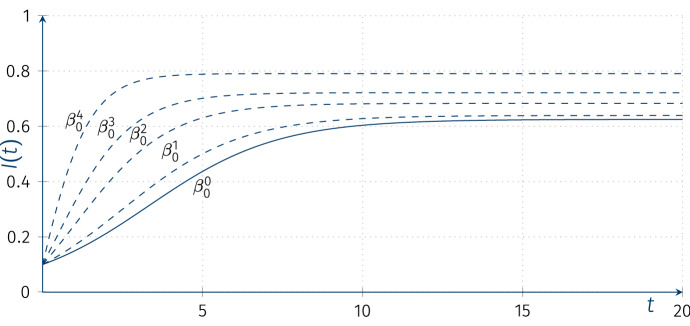
“External” SIS model solutions for different (deterministic) parameters $\beta _0$: $\beta _0^0=0$ (classical SIS), $\beta _0^1=0.02$, $\beta _0^2=0.1$, $\beta _0^3=0.2$, $\beta _0^4=0.5$; $\beta _1=0.8$, $\alpha =0.3$, $I(0)=0.1$

Figure 5 shows the solutions of “external” SIS models for various values of the parameter $\beta _0$.

It can be proved that conditions ([Disp-formula Equ1]) and $(\bigstar _M)$ hold for the SIS model as the phase space is compact and

$$\begin{aligned} \sup _{t, x} \{ |S'(t)| , |I'(t)| \} < \infty \end{aligned}$$

($t \ge 0$ and $x\in [0,1]$ is an initial condition). Condition ([Disp-formula Equ1]) holds from the mentioned boundedness of derivatives. On the other hand, the condition $(\bigstar _M)$ can be derived from the SIS master equations (Eq. 4.1), and our assumption that $S + I \equiv \mathrm {const} ( = 1 )$. Namely, we have

$$\begin{aligned}| I_t( p) - I_t(q) | \le |p - q|\mathrm{e}^{\gamma t} . \end{aligned}$$

Hence,

$$\begin{aligned} \begin{aligned} |S_t(x) - S_t(y) |&= |(1 - I_t(1 - x)) - (1 - I_t(1 - y)) | = |I_t(1 - x ) - I_t(1-y)| \\&\le |(1- x) - (1-y)|\mathrm{e}^{\gamma t} = |x-y|\mathrm{e}^{\gamma t} \ . \end{aligned} \end{aligned}$$

Similarly to the previous stochastic modification we implement the “external” rate $\beta _0$ as a CTHMC with intensity matrix *Q* on the finite state space $\{0,1,\ldots ,M\}$. Assume that the external incidence rate $\beta _0=\beta _0(t)$ is the realization of a CTHMC on the state space $\{0, 0.044, 0.11, 0.01\}$ with intensity matrix *Q* (as previously) and $P(\beta (0)=0)=1$. The other parameters for SIS model are as follows: $I(0)=0.01, \beta _1=0.0836,\alpha =0.05, K=1$.

**Fig. 6:**
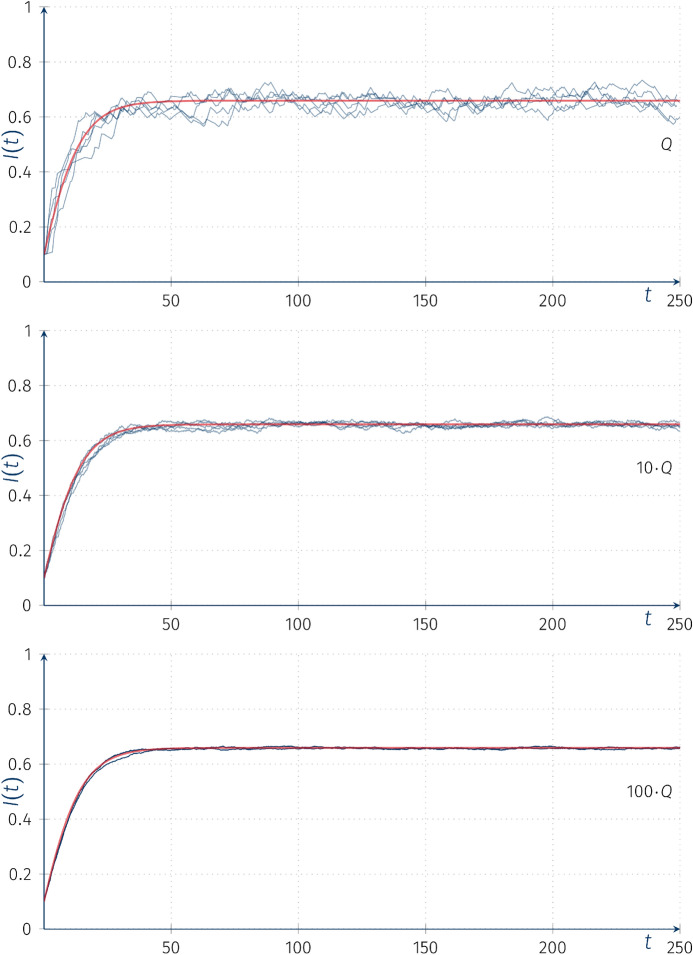
Trajectories of the piecewise-deterministic Markov processes $I(t)(\omega )$ for SIS models with random coefficients with “external” incidence rate models with intensity matrices *Q*, 10*Q*, 100*Q*, red line denotes the deterministic solution of the ODE with parameter $\overline{\beta _0}$

Figure 6 shows the trajectories of the process $I(t)(\omega )$ superimposed on the ordinary deterministic solution *I*(*t*) with parameter $\overline{\beta _0}$ corresponding to $\mu _*\circ (0, 0.044, 0.11, 0.01)$. Again we find that with multiplied intensity matrices *kQ* we can observe the convergence to the deterministic $\overline{\beta _0}$ solution as $k\rightarrow \infty$, i.e. for high-frequency changes the in the underlying driving process, the observed process looks smooth.

**Fig. 7:**
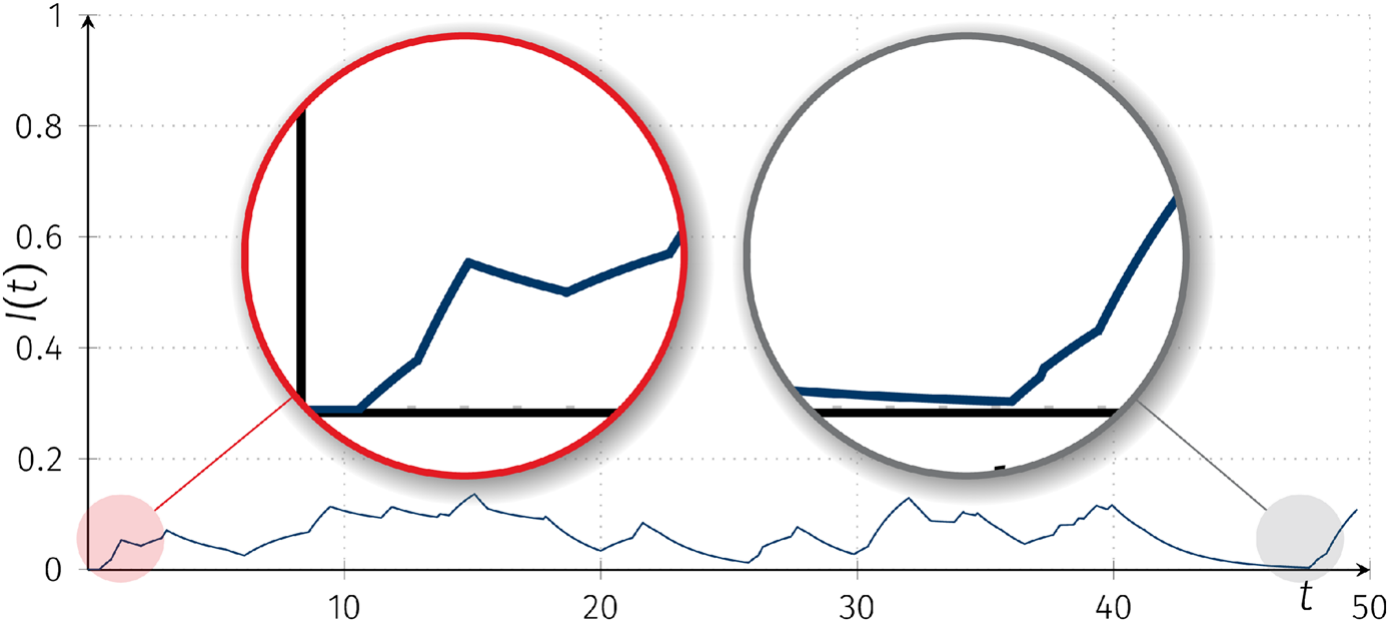
Trajectories of the piecewise-deterministic Markov processes $I(t)(\omega )$ for for SIS models with random coefficients with “external” incidence rate models with intensity matrices *Q*, $\beta _0(0)=0$, $\beta _1=0.01$, $\alpha =0.05$, $I(0)=0$, $K=1$

From the formulation of the classical SIS model, assuming that at any given time *t* the number of infected individuals *I*(*t*) equals 0, we obtain a stable trivial equilibrium. However, it is not the case in our new model, as the positive “external” incidence rate makes it unstable. Figure 7 shows a sample trajectory presenting the origination of epidemics from a population free from infected individuals.

## Worked example: the SIR model with random coefficients

Similarly to the SIS (Sect. 4) models, we introduce an external stochastic infectious mechanism to the classical SIR model. We are unable to find analytical closed form solutions. The master equation of the SIR model guarantees that the generated by it semiflows allow for stochastic extensions. In order to obtain that the corresponding, controlled by a CTHMC, semiflows are stochastic processes, we simply explain that the crucial moment of the proof of our Thm 2 is the step where we show that the mapping

$$\begin{aligned} \mathfrak {H}_{r;m} \ni \xi _{\circ }(\omega ) \mapsto \varPhi _r^{\xi _{\circ }(\omega )}(x) \in X \end{aligned}$$

is $(d_r, \varrho )$ continuous (hence measurable). Now the continuity will follow from the boundedness of derivatives $\sup \{ |S'(t)| , |I'(t)|, |R'(t) | \} < \infty$. Let us add that the corresponding vector fields on $[0,1]\times [0,1] \times [0,1]$ are furthermore smooth. Therefore, if the trajectories $\xi _{\circ }(\omega )$ are close in the metric $d_r$ on $\mathfrak {H}_{r;m}$ then the final states (i.e. the ends of paths) $\varPhi _r^{\xi _{\circ }(\omega )}(x)$ are also close.

Let us stress that this section is strongly supported by numerics, instead of being a purely mathematical solution of the modified SIR model. As long as there is no explicit closed form solution it may be difficult to find (or estimate) the expansiveness of a semiflow. We can show that if $x > y$ then $| S_t(x) - S_t(y) | \le |x\mathrm{e}^{-\beta _0t} - y \mathrm{e}^{-(\beta _0 + \beta _1 )t} |$. The expansiveness of $I_t(p)$ or $R_t(r)$ may be even more complicated.

Similarly to the SIS (Sect. 4) models, we introduce an external stochastic infectious mechanism to the classical SIR model. We again remind the reader that we have a normalized population size, $N=1$.

5.1 $$\begin{aligned} \left\{ \begin{aligned} \frac{\mathrm{d}S(t)}{\mathrm{d}t}&= -\beta _1 S(t)\ I(t) -\beta _0 S(t) \\ \frac{\mathrm{d}I(t)}{\mathrm{d}t}&=\ \beta _1 S(t)\ I(t) +\beta _0 S(t) - {\alpha }I(t),\\ \frac{\mathrm{d}R(t)}{\mathrm{d}t}&=\ {\alpha }I(t), \end{aligned}\right. \end{aligned}$$

where for all $t>0$, $S(t)+I(t)+R(t)=1\equiv \text {const.}$ If $\beta _0=0$, then we obtain the classical SIR model. In the classical case it can be shown that $\lim _{t\rightarrow \infty } I(t)=0$, and that the limits of *S*(*t*) and *R*(*t*) (as $t\rightarrow \infty$) are finite but depend on the initial conditions. Assuming $\beta _0=0$, if the effective reproduction number

$$\begin{aligned} \varrho = R_0S(0)=S(0)\frac{\beta }{\alpha }\end{aligned}$$

satisfies $\varrho \le 1$, then there is no epidemic, and *I*(*t*)’s decrease to zero is monotonic. However, if $\varrho >1$, then *I*(*t*) first increases before decreasing to zero, a situation which we call an epidemic.

Following the procedure used in Harko et al. (2014) we will now turn to analyzing the SIR model with external infection. We will not obtain a closed-form analytical solution, but reduce it to an Abel equation of the first kind. It follows from Eq. (5.1) that

5.2 $$\begin{aligned} \frac{S'}{S}=-\beta _0 -\beta _1 I. \end{aligned}$$

Hence

5.3 $$\begin{aligned} I&=-\frac{\beta _0 }{\beta _1 }-\frac{1}{\beta _1 }\frac{S'}{S}, \end{aligned}$$

and

5.4 $$\begin{aligned} -\beta _1 I'&= \frac{S''}{S}-\left( \frac{S'}{S}\right) ^2. \end{aligned}$$

Similarly from Eqs. (5.1) and (5.3)

5.5 $$\begin{aligned} I'&=(\beta _0 +\beta _1 I)S-{\alpha }I=-S'-{\alpha }I, \end{aligned}$$

5.6 $$\begin{aligned} I'&=-S'-{\alpha }\left( -\frac{\beta _0 }{\beta _1 }-\frac{1}{\beta _1 }\frac{S'}{S}\right) . \end{aligned}$$

Combining Eqs. (5.4) and (5.6) we obtain

5.7 $$\begin{aligned}&\frac{S''}{S}-\left( \frac{S'}{S}\right) ^2=\beta _1 S'-\beta _0 {\alpha }-{\alpha }\frac{S'}{S}, \end{aligned}$$

5.8 $$\begin{aligned}&\frac{S''}{S}-\left( \frac{S'}{S}\right) ^2+{\alpha }\frac{S'}{S}-\beta _1 S'+\beta _0 {\alpha }=0. \end{aligned}$$

From Eq. (5.1), solving for *R*(*t*), we obtain

5.9 $$\begin{aligned} R'&=-\frac{{\alpha }}{\beta _1 }\left( \beta _0 +\frac{S'}{S}\right) =-\frac{{\alpha }\beta _0 }{\beta _1 }-\frac{{\alpha }}{\beta _1 }\frac{S'}{S}, \end{aligned}$$

thus

5.10 $$\begin{aligned} S(t)&=S(0)\ e^{-\frac{\beta _1 }{{\alpha }}\left( R(t)-R(0)\right) -\beta _0 t} \end{aligned}$$

or (in short)

5.11 $$\begin{aligned} S(t)&=D\ e^{-\frac{\beta _1 }{{\alpha }}R(t)-\beta _0 t}, \text { where }D=S(0)\ e^{\frac{\beta _1 }{{\alpha }}R(0)}. \end{aligned}$$

Differentiating Eq. (5.11)

5.12 $$\begin{aligned} S'&=D\left( -\frac{\beta _1 }{{\alpha }}R'-\beta _0 \right) \ e^{-\frac{\beta _1 }{{\alpha }}R(t)-\beta _0 t}=-\left( \frac{\beta _1 }{{\alpha }}R'+\beta _0 \right) \ S, \end{aligned}$$

5.13 $$\begin{aligned} \frac{S'}{S}&=-\left( \frac{\beta _1 }{{\alpha }}R'+\beta _0 \right) . \end{aligned}$$

Hence

5.14 $$\begin{aligned} R'&=-\frac{{\alpha }}{\beta _1 }\left( \beta _0 +\frac{S'}{S}\right) \end{aligned}$$

and

5.15 $$\begin{aligned} R''&=-\frac{{\alpha }}{\beta _1 }\left( \frac{S'}{S}\right) '=-\frac{{\alpha }}{\beta _1 }\left( \frac{S''}{S}-\left( \frac{S'}{S}\right) ^2\right) . \end{aligned}$$

Substituting Eqs. (5.12)–(5.15) into Eq. (5.8), and after reordering, we obtain

5.16 $$\begin{aligned} R''=-{\alpha }R'+{\alpha }\ D\left( \beta _0 +\frac{\beta _1 }{{\alpha }}R'\right) \ e^{-\frac{\beta _1 }{{\alpha }}R-\beta _0 t}. \end{aligned}$$

Let

5.17 $$\begin{aligned} u(t)=e^{-\frac{\beta _1 }{{\alpha }}R(t)-\beta _0 t} \end{aligned}$$

and as

5.18 $$\begin{aligned} u'&=-\left( \beta _0 +\frac{\beta _1 }{{\alpha }}R'\right) \ u \end{aligned}$$

5.19 $$\begin{aligned} \frac{u'}{u}&=-\beta _0 -\frac{\beta _1 }{{\alpha }}R' \end{aligned}$$

we obtain

5.20 $$\begin{aligned} R'&=-\frac{{\alpha }}{\beta _1 }\left( \beta _0 +\frac{u'}{u}\right) \end{aligned}$$

and

5.21 $$\begin{aligned} R''&=-\frac{{\alpha }}{\beta _1 }\left( \frac{u'}{u}\right) '=-\frac{{\alpha }}{\beta _1 }\left( \frac{u''}{u}-\left( \frac{u'}{u}\right) ^2\right) . \end{aligned}$$

Substituting Eqs. (5.20) and (5.21) into Eq. (5.16) we obtain

5.22 $$\begin{aligned} u\ u''-(u')^2+\left( {\alpha }-\beta _1 \ D u\right) u\ u'+{\alpha }\beta _0 u^2=0. \end{aligned}$$

Let $$\varPhi (u(t))=t$$, i.e. $$\varPhi =u^{-1}$$,

5.23 $$\begin{aligned} \left( u^{-1}(t)\right) '=\left( \varPhi (u)\right) '=\frac{1}{u'(t)}=\frac{1}{u'\left( \varPhi (u(t))\right) }, \end{aligned}$$

thus

5.24 $$\begin{aligned} \frac{\mathrm{d}t}{\mathrm{d}u}=\frac{1}{u'\left( \varPhi (u)\right) }. \end{aligned}$$

Applying the chain rule, and from Eq. (5.23)

5.25 $$\begin{aligned} \varPhi ''(u)=-\frac{u''(\varPhi (u))\ \varPhi '(u)}{\left( u'\left( \varPhi (u)\right) \right) ^2}=-\frac{u''(\varPhi (u))}{\left( u'\left( \varPhi (u)\right) \right) ^3}. \end{aligned}$$

Let $\phi (u)=\varPhi '(u)$, then $\phi '(u)=\varPhi ''(u)$ and

5.26 $$\begin{aligned} u'(\varPhi (u(t)))&=\frac{1}{\phi (u(t))}, \end{aligned}$$

5.27 $$\begin{aligned} u''(\varPhi (u(t)))&=-\phi '(u(t))\cdot \left( u'\left( \varPhi (u)\right) \right) ^{-3}=-\frac{\phi '(u(t))}{\phi ^3(u(t))}. \end{aligned}$$

Substituting into Eq. (5.22) we obtain

5.28 $$\begin{aligned} u\cdot \left[ -\frac{\phi '(u)}{\phi ^3(u)}\right] -\frac{1}{\phi ^2(u)}+ \left( {\alpha }-\beta _1 D u\right) \ u\ \frac{1}{\phi (u)}+ {\alpha }\beta _0 u^2=0. \end{aligned}$$

Multiplying by $-\frac{\phi ^3(u)}{u}$ we obtain

5.29 $$\begin{aligned} -\phi '(u) -\frac{\phi (u)}{u}+ \left( {\alpha }-\beta _1 D u\right) \ \phi ^2(u)+ {\alpha }\beta _0 u\ \phi ^3(u)=0 \end{aligned}$$

or in an equivalent form

5.30 $$\begin{aligned} \phi '(u)={\alpha }\beta _0 u\ \phi ^3(u)+\left( {\alpha }-\beta _1 D u\right) \ \phi ^2(u) -\frac{1}{u}\phi (u). \end{aligned}$$

We can recognize Eq. (5.30) as an Abel equation of the first kind, provided that ${\alpha }\beta _0 u\ne 0$ (§1.4.1 Polyanin and Zaitsev (1995)). If ${\alpha }\beta _0 u=0$ the equation reduces to a Riccati-type equation, or if ${\alpha }-\beta _1 D u=0$ it reduces to a Bernoulli-type equation.

Equation (5.30) has no closed-form analytical solution. Introducing $\beta _0>0$ to the SIR model results in a substantial change in our simulation process. We will rely on the R’s (R Core Team 2020) package deSolve to solve the SIR ODE system, Eq. (5.1), between the interarrival times of the CTHMC.

**Fig. 8:**
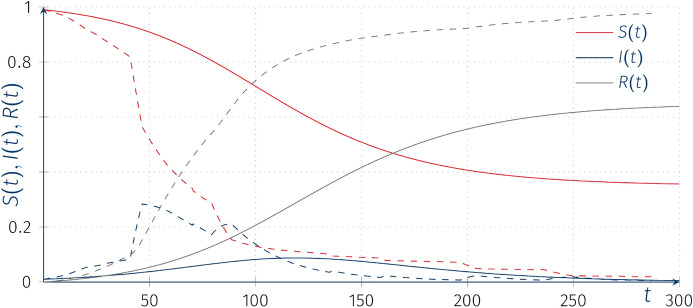
Trajectories of the piecewise-deterministic Markov model (dashed lines), $SIR(t)(\omega )$ for the “external” incidence rate models with intensity matrices 0.1*Q*, $\beta _0(0)=0.001$, $\beta _1=0.08$, $\alpha =0.05$, $I(0)=0.01$, $R(0)=0$; solid lines represent the solution of the classical SIR model (with $\beta _0\equiv 0$)

After introducing the “external” transmission rate $\beta _0>0$, we obtain only the trivial asymptotic equilibrium $(S^\star ,I^\star ,R^\star )=(0,0,1)$. This is as all susceptible individuals will become eventually infected either via contact with the infected and infectious individuals (assuming $I(t)>0$) or through the “external” transmission. Allowing for $\beta _0$ to be described as a value of CTHMC provide us with a framework in which short “impulses” may switch the behavior of the solution $(I(t), t>0)$ between consecutive growth stages before decreasing to zero along the solution $(S(t), t>0)$. Figure 8 shows a sample trajectories of SIR solutions assuming that $\beta _0=\beta _0(t)$ is a Markov process with intensity matrix 0.1*Q* (for long inter-arrival times of the process) on a finite state space $\{0.001,0.002,0,0.05\}$. We can clearly observe consecutive “waves” of the epidemic.

**Fig. 9:**
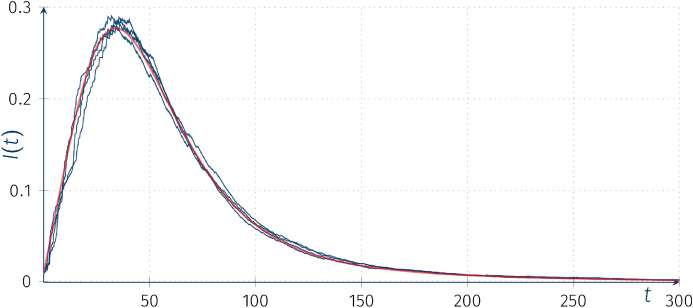
Trajectories of the piecewise-deterministic Markov processes $I(t)(\omega )$ for SIR models with random coefficients with the “external” incidence rate SIR model with intensity matrix 10*Q*, $\beta _0(0)=0$, $\beta _1=0.08$, $\alpha =0.05$, $I(0)=0.01$, $R(0)=0$; red line denotes the deterministic solution of the ODE with parameter $\overline{\beta _0}$

In line with our previous observations we can see that with the increase of the intensity matrix of the CTHMC *kQ*, as $k\rightarrow \infty$, the trajectories converge to the deterministic solution with parameter $\overline{\beta _0}$ corresponding to $\overline{\beta _0}\equiv {\text {E}}\left[ \beta _0 \right] =\mu _*\circ (0.001,0.002,0,0.05)$ (see Fig. 9).

## Discussion and conclusions

We end with a few historical remarks. The topic we presented is not new. The long-term behaviour of dynamical systems subjected to random perturbations was studied from different points of view (theoretical or practical computer simulations). It is quite impossible to give representative references, but we simply mention a few of them (see Benaïm and Strickler 2019; Chen-Charpentier and Stanescu 2010; Gray et al. 2012; Rami et al. 2014; Roberts 2017). The common features of these models is that the dynamics is randomly interrupted so that the internal (very often hidden) state of the systems is changed, according to a law that depends on the state just before this intervention. In our work, the duration of the time intervals between interventions are independent and have exponential distributions (Markov models).

Describing the asymptotic behaviour of trajectories of randomly controlled semiflows seems to be a difficult task. There is no hope for achieving explicit analytical formulæ, in particular, not even for deterministic SIR models, see Sect. 5. On the other hand, these notions provide theoretical models, which are recently a subject of interest for many research groups. In our work we employ computer techniques and find, by simulations, their statistical properties. The main goal is to find the dependence of these statistics for growing frequencies of the control processes (e.g. potential social contacts). This is reflected by considering sequences *kQ* of intensity matrices, where $k\rightarrow \infty$. For larger *k* the intensity matrix *kQ* will exhibit higher frequencies of change in the steering process. For special classes (in particular for SIR semiflows) the randomness of $\varPhi ^{\xi _{\circ }(\omega )}_t(x) = \zeta _t (x, \omega )$ finally ($k\rightarrow \infty$) will be extinguished, approaching a deterministic semiflow for some specific mean $\vartheta _\diamond \in \varTheta$ (not necessarily in $\varTheta^{*}$).

For simplicity we considered control Markov processes $\varXi$ that are **irreducible** on finite $\varTheta^{*}$. It follows that it has a unique stationary distribution $\mu _*$ on $\varTheta^{*}$ (see Allen (2011), Norris (2007), p. 118). By the ergodic theorem (see Norris (2007), p. 126) the trajectories $\xi _t(\omega )$ with probability 1 visit each fixed state $\vartheta \in \varTheta^{*}$ with frequency $\mu _*(\vartheta )$. If the intensity coefficient $k\rightarrow \infty$, then on each (even very short) time interval $(r,s) \subset [0, \infty )$ the control sequence $^{(k)}\xi _t(\omega )$ switches parameters $\vartheta \in \varTheta^{*}$ accordingly to the vector $\mu _*$ and many times (more and more when *k* is large), producing in some sense an averaged parameter $\vartheta _{\diamond } = \sum _{\vartheta \in \varTheta^{*}} \vartheta \mu _*(\vartheta )$. We notice that for $\varTheta^{*} = \{ 0, 1, 2,\ldots ,M \}$ the expected value $\vartheta _{\diamond }$ generally does not belong to $\varTheta^{*}$ any more. Therefore, the proposed approach is not universal, sometimes the parameters $\vartheta$ are not even numerical. In the considered SIS/SIR models, the parameters $\beta _0 (= \xi _{\circ }(\omega )) \in [0,M]$ behave in a linear (affine) fashion. Hence, one may evaluate the average $\vartheta _{\diamond }$ and ask whether the asymptotic behaviour (i.e. when $k\rightarrow \infty$) of the stochastic dynamics is carried over.

We used computer simulations to look at the high-frequency SIR properties. Namely, it looks like with growing frequencies the random trajectories become smoother and the total variance of the process decays to 0 and we seem to arrive at almost smooth graphs, characteristic for deterministic models. Hence, we can hypothesize that the high frequencies, i.e. short transition times, of the control process result in almost sure convergence of the controlled dynamical system.

Let us finally comment that the high-frequency is imitated here by a direct increase of the intensity matrix, *kQ*. Of course, it would be desirable to work with more general approaches, e.g. considering $kQ_k$, where for every *k* we only impose $\mu _*\circ Q_k = 0$. This is a direction for further theoretical work but here we dedicated to it a short, connected to the current spread of SARS-CoV-2 virus, Monte Carlo study. Interestingly, very recently a SIRD (SIR and Death) with random coefficients model was considered for describing the COVID-19 dynamics in Italy (Alòs et al. 2020). There they considered a fractional Brownian motion process for the infection parameter, $\beta$ in Eqs. (4.1) and (5.1). It is worth pointing out a key difference between modelling via a fractional Brownian motion and the random dynamical system approach presented here. A fractional Brownian motion in general will not have piecewise smooth (differentiable) trajectories. On the other hand, a random dynamical system, as described in this work, will.

## Figures and Tables

**Fig. 10:**
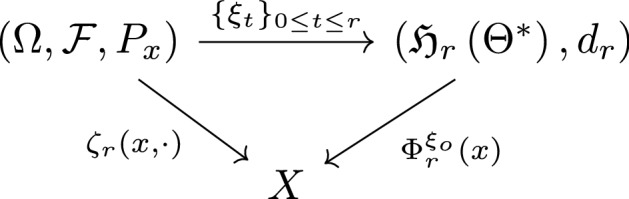
Illustration of the mapping $\zeta _r(x, \omega )$

## Appendix: Proofs of Lemmata and Theorems from Sect. 2

### Proof of Lemma 2

The functions $\mathfrak {h}, \mathfrak {g} \in \mathfrak {H}(\varTheta^{*})$ have finitely many jumps on the considered interval [0, *r*]. We recall that the total length of the intervals $(w_l, w_{l+1})$ on which the functions $\mathfrak {h} , \mathfrak {g}$ differ is $d_r(\mathfrak {h}, \mathfrak {g})$ (we are confined to the interval [0, *r*] only, thus the point $w_{l+1}$ at the end of the final interval should be understood as *r*).

Case I ($n_* = 2j$). It follows from the condition ([Disp-formula Equ2]) that

$$\begin{aligned} \varrho (\varPhi ^{\mathfrak {h}}_{w_0}(x), \varPhi ^{\mathfrak {g}}_{w_0}(y)) \le \varDelta (w_0)\varrho (x, y) \end{aligned}$$

as $$\mathfrak {h}(t) = \mathfrak {g}(t)$$ for all $$t\in [0, w_0]$$ (we remind again that if $$w_0 \ge r$$, then $$ \varrho (\varPhi ^{\mathfrak {h}}_r(x), \varPhi ^{\mathfrak {g}}_r(y)) \le \varDelta (r)\varrho (x, y))$$). On the interval $$[w_0, w_1)$$ the functions $$\mathfrak {h}$$ and $$\mathfrak {g}$$ differ, so applying condition ([Disp-formula Equ1]) we obtain (the constant *L* corresponds to $$T=r$$)

$$\begin{aligned} \begin{aligned}&\varrho (\varPhi ^{\mathfrak {h}}_{w_1}(x) ,\varPhi ^{\mathfrak {g}}_{w_1}(y) ) \le \varrho (\varPhi ^{\mathfrak {h}}_{w_1}(x) ,\varPhi ^{\mathfrak {h}}_{w_0}(x) ) + \varrho ( \varPhi ^{\mathfrak {h}}_{w_0}(x) ,\varPhi ^{\mathfrak {g}}_{w_0}(y) ) + \varrho (\varPhi ^{\mathfrak {g}}_{w_0}(y) ,\varPhi ^{\mathfrak {g}}_{w_1}(y) ) \\&\quad \le 2L(w_1-w_0) + \varrho (\varPhi ^{\mathfrak {h}}_{w_0}(x), \varPhi ^{\mathfrak {g}}_{w_0}(y) ) \le 2L(w_1-w_0) + \varDelta (w_0) \varrho (x,y) . \end{aligned} \end{aligned}$$

Substituting $0 \leftarrow w_1$, $w_0 \leftarrow w_2$, $\varPhi ^{\mathfrak {h}}_{w_1}(x) \leftarrow x$, and $\varPhi ^{\mathfrak {g}}_{w_1}(y) \leftarrow y$ we obtain

$$\begin{aligned} \begin{aligned}&\varrho ( \varPhi ^{\mathfrak {h}}_{w_2}(x) ,\varPhi ^{\mathfrak {g}}_{w_2}(y)) \le \varDelta (w_2 - w_1) \varrho (\varPhi ^{\mathfrak {h}}_{w_1}(x) ,\varPhi ^{\mathfrak {g}}_{w_1}(y)) \\&\quad \le \varDelta (w_2 - w_1)(2L(w_1-w_0) + \varDelta (w_0) \varrho (x,y) ) \\&\quad = 2L(w_1 - w_0)\varDelta (w_2 - w_1) + \varDelta (w_2 - w_1)\varDelta (w_0)\varrho (x,y)\ . \end{aligned} \end{aligned}$$

Similarly we have

$$\begin{aligned} \begin{aligned}&\varrho ( \varPhi ^{\mathfrak {h}}_{w_3}(x) ,\varPhi ^{\mathfrak {g}}_{w_3}(y) ) \\&\quad = \varrho (\varPhi ^{\mathfrak {h}(\cdot + w_1)}_{w_3 - w_1}(\varPhi ^{\mathfrak {h}}_{w_1}(x) ) ,\varPhi ^{\mathfrak {g}(\cdot + w_1)}_{w_3-w_1}(\varPhi ^{\mathfrak {g}}_{w_1}(y) ) ) \\&\quad \le 2L(w_3 -w_2 ) + \varDelta (w_2 - w_1)\varrho ( \varPhi ^{\mathfrak {h}}_{w_1}(x) ,\varPhi ^{\mathfrak {g}}_{w_1}(y) ) \\&\quad \le 2L(w_3 - w_2) + \varDelta (w_2 - w_1)( 2L(w_1-w_0) + \varDelta (w_0) \varrho (x,y) ) \\&\quad = 2L\left( (w_3 - w_2) + (w_1 - w_0)\varDelta (w_2 - w_1) \right) + \varDelta (w_2 - w_1)\varDelta (w_0)\varrho (x,y) . \end{aligned} \end{aligned}$$

Iterating this procedure we finally obtain

$$\begin{aligned} \begin{aligned}&\varrho (\varPhi ^{\mathfrak {h}}_{w_{2j+1}}(x) , \varPhi ^{\mathfrak {g}}_{w_{2j+1}}(y)) \\&\quad \le 2L ( (w_{2j+1} - w_{2j}) + (w_{2j-1} - w_{2j-2})\varDelta (w_{2j } - w_{2j-1}) \\&\qquad + (w_{2j-3} - w_{2j-4})\varDelta (w_{2j} - w_{2j-1})\varDelta (w_{2j-2} - w_{2j-3}) + \cdots \\&\qquad + (w_1 - w_0)\varDelta (w_{2j } - w_{2j-1}) + (w_{2j-3} - w_{2j-4})\varDelta (w_{2j} - w_{2j-1})\cdots \varDelta (w_2 - w_1) ) \\&\qquad + \varDelta (w_{2j } - w_{2j-1})\varDelta (w_{2j-2} - w_{2j-3})\cdots \varDelta (w_{2} - w_{1})\varDelta (w_0 - 0)\varrho (x,y) \ . \end{aligned} \end{aligned}$$

This bound must be obviously confined to [0, *r*] , so we in fact can obtain

$$\begin{aligned} \begin{aligned}&\varrho (\varPhi ^{\mathfrak {h}}_{r}(x) , \varPhi ^{\mathfrak {g}}_{r}(y)) \\&\quad \le 2L ( (r - w_{2j}) + (w_{2j-1} - w_{2j-2})\varDelta (w_{2j } - w_{2j-1}) \\&\qquad + (w_{2j-3} - w_{2j-4})\varDelta (w_{2j} - w_{2j-1})\varDelta (w_{2j-2} - w_{2j-3}) + \cdots \\&\qquad + (w_1 - w_0)\varDelta (w_{2j } - w_{2j-1}) + (w_{2j-3} - w_{2j-4})\varDelta (w_{2j} - w_{2j-1})\cdots \varDelta (w_2 - w_1) ) \\&\qquad + \varDelta (w_{2j } - w_{2j-1})\varDelta (w_{2j-2} - w_{2j-3})\cdots \varDelta (w_{2} - w_{1})\varDelta (w_0 - 0)\varrho (x,y) \ . \end{aligned} \end{aligned}$$

Case II, $$n_* = 2j-1$$

$$\begin{aligned} \begin{aligned}&\varrho (\varPhi ^{\mathfrak {h}}_{w_{2j}}(x) , \varPhi ^{\mathfrak {g}}_{w_{2j}}(y)) \\&\quad \le 2L ((w_{2j-1} - w_{2j-2})\varDelta (w_{2j} - w_{2j-1}) \\&\qquad + (w_{2j-3} - w_{2j-4})\varDelta (w_{2j} - w_{2j-1}) \varDelta (w_{2j-2} - w_{2j-3}) \\&\qquad + (w_{2j-5} - w_{2j-6})\varDelta (w_{2j} - w_{2j-1}) \varDelta (w_{2j-2} - w_{2j-3}) \varDelta (w_{2j-4} - w_{2j-5}) + \cdots \\&\qquad + (w_1 - w_0)\varDelta (w_{2j} - w_{2j-1}) \varDelta (w_{2j-2} - w_{2j-3}) \cdots \varDelta (w_{2} - w_{1})) \\&\qquad + \varDelta (w_{2j} - w_{2j-1}) \varDelta (w_{2j-2} - w_{2j-3}) \cdots \varDelta (w_{2} - w_{1})\varDelta (w_0 - 0)\varrho (x,y) \ . \end{aligned} \end{aligned}$$

Taking $$ t = r$$ we obtain

$$\begin{aligned} \begin{aligned}&\varrho (\varPhi ^{\mathfrak {h}}_r (x) , \varPhi ^{\mathfrak {g}}_r(y)) \\&\quad \le 2L ((w_{2j-1} - w_{2j-2})\varDelta (r - w_{2j-1}) \\&\qquad + (w_{2j-3} - w_{2j-4})\varDelta (r - w_{2j-1}) \varDelta (w_{2j-2} - w_{2j-3}) \\&\qquad + (w_{2j-5} - w_{2j-6})\varDelta (r - w_{2j-1}) \varDelta (w_{2j-2} - w_{2j-3}) \varDelta (w_{2j-4} - w_{2j-5}) + \cdots \\&\qquad + (w_1 - w_0)\varDelta (r - w_{2j-1}) \varDelta (w_{2j-2} - w_{2j-3}) \cdots \varDelta (w_{2} - w_{1})) \\&\qquad + \varDelta (r - w_{2j-1}) \varDelta (w_{2j-2} - w_{2j-3}) \cdots \varDelta (w_{2} - w_{1})\varDelta (w_0 - 0)\varrho (x,y) \ . \end{aligned} \end{aligned}$$

$$\blacksquare $$

### Proof of Lemma 3

If $w_1 \ge r$ (we apply conditions ([Disp-formula Equ2]) and ([Disp-formula Equ1])), then

$$\begin{aligned} \begin{aligned}&\varrho (\varPhi ^{\mathfrak {h}}_r(x) ,\varPhi ^{\mathfrak {g}}_r(y) ) \le \varrho (\varPhi ^{\mathfrak {h}}_r(x) , \varPhi ^{\mathfrak {h}}_0(x) ) \\&\quad + \varrho (\varPhi ^{\mathfrak {h}}_0(x) , \varPhi ^{\mathfrak {g}}_0(y) ) + \varrho (\varPhi ^{\mathfrak {g}}_0(y) , \varPhi ^{\mathfrak {g}}_r(y) ) \le 2Lr + \varrho (x,y) \ . \end{aligned} \end{aligned}$$

Case I. Let $n_* = 2j$. Reasoning as before we obtain $\varrho (\varPhi ^{\mathfrak {h}}_{w_1}(x) ,\varPhi ^{\mathfrak {g}}_{w_1}(y) ) \le 2Lw_1 + \varrho (x,y)$. Starting from $t = w_1$ we may apply the previous lemma as $\mathfrak {h}(w_1) = \mathfrak {g}(w_1)$. We simply replace *r* by $r - w_1$ and have

$$\begin{aligned} \begin{aligned}&\varrho (\varPhi ^{\mathfrak {h}}_r(x) ,\varPhi ^{\mathfrak {g}}_r(y) ) = \varrho (\varPhi ^{\mathfrak {h}(\cdot + w_1)}_{r-w_1}(\varPhi ^{\mathfrak {h}}_{w_1}(x)) , \varPhi ^{\mathfrak {g}(\cdot + w_1)}_{r-w_1}(\varPhi ^{\mathfrak {g}}_{w_1}(y))) \\&\quad \le 2L( (r - w_{2j}) + (w_{2j-1} - w_{2j-2})\varDelta (w_{2j} - w_{2j-1}) \\&\qquad + (w_{2j-3} - w_{2j-4} )\varDelta (w_{2j} - w_{2j-1})\varDelta (w_{2j-2} - w_{2j-3}) + \cdots \\&\qquad + (w_3 - w_2)\varDelta (w_{2j} - w_{2j-1})\varDelta (w_{2j-2} - w_{2j-3})\cdots \varDelta (w_4 - w_3) ) \\&\qquad + \varDelta (w_{2j} - w_{2j-1})\varDelta (w_{2j-2} - w_{2j-3})\cdots \varDelta (w_4 - w_3)\varDelta (w_2 - w_1)(2Lw_1 + \varrho (x,y)) \\&\quad = 2L( (r - w_{2j}) + (w_{2j-1} - w_{2j-2})\varDelta (w_{2j} - w_{2j-1}) \\&\qquad + (w_{2j-3} - w_{2j-4} )\varDelta (w_{2j} - w_{2j-1})\varDelta (w_{2j-2} - w_{2j-3}) + \cdots \\&\qquad + (w_3 - w_2)\varDelta (w_{2j} - w_{2j-1})\varDelta (w_{2j-2} - w_{2j-3})\cdots \varDelta (w_4 - w_3) \\&\qquad + (w_1 - 0))\varDelta (w_{2j} - w_{2j-1})\varDelta (w_{2j-2} - w_{2j-3})\cdots \varDelta (w_4 - w_3)\varDelta (w_2 - w_1) \\&\qquad + \varDelta (w_{2j} - w_{2j-1})\varDelta (w_{2j-2} - w_{2j-3})\cdots \varDelta (w_4 - w_3)\varDelta (w_2 - w_1)\varrho (x,y) \ . \end{aligned} \end{aligned}$$

Case II. $n_* = 2j-1$. We begin as above

$$\begin{aligned} \begin{aligned}&\varrho (\varPhi ^{\mathfrak {h}}_r(x) ,\varPhi ^{\mathfrak {g}}_r(y) ) = \varrho (\varPhi ^{\mathfrak {h}(\cdot + w_1)}_{r-w_1}(\varPhi ^{\mathfrak {h}}_{w_1}(x)) , \varPhi ^{\mathfrak {g}(\cdot + w_1)}_{r-w_1}(\varPhi ^{\mathfrak {g}}_{w_1}(y))) \\&\quad \quad \le 2L( (w_{2j-1} - w_{2j-2})\varDelta (r - w_{2j-1}) \\&\qquad + (w_{2j-3} - w_{2j-4})\varDelta (r - w_{2j-1})\varDelta (w_{2j-2} - w_{2j-3}) + \cdots \\&\qquad + (w_3 - w_2)\varDelta (r - w_{2j-1})\varDelta (w_{2j-2} - w_{2j-3})\cdots \varDelta (w_4 - w_3) ) \\&\qquad + \varDelta (r - w_{2j-1})\varDelta (w_{2j-2} - w_{2j-3})\cdots \varDelta (w_4 - w_3)\varDelta (w_2 - w_1)(2Lw_1 + \varrho (x,y)) \\&\qquad = 2L( (w_{2j-1} - w_{2j-2})\varDelta (r - w_{2j-1}) \\&\qquad + (w_{2j-3} - w_{2j-4})\varDelta (r - w_{2j-1})\varDelta (w_{2j-2} - w_{2j-3}) + \cdots \\&\qquad + (w_3 - w_2)\varDelta (r - w_{2j-1})\varDelta (w_{2j-2} - w_{2j-3})\cdots \varDelta (w_4 - w_3) \\&\qquad + (w_1 - 0)\varDelta (r - w_{2j-1})\varDelta (w_{2j-2} - w_{2j-3})\cdots \varDelta (w_4 - w_3)\varDelta (w_2 - w_1) ) \\&\qquad + \varDelta (r - w_{2j-1})\varDelta (w_{2j-2} - w_{2j-3})\cdots \varDelta (w_4 - w_3)\varDelta (w_2 - w_1)\varrho (x,y) . \end{aligned} \end{aligned}$$

$$\blacksquare $$

### Proof of Theorem 2

We must show that for each fixed $r\in [0, \infty )$ the mapping $\varOmega \ni \omega \mapsto \zeta _r(x, \omega ) \in X$ is measurable. We illustrate this situation in Fig. 10.

Let us define

$$\begin{aligned} \begin{aligned} \varOmega _{r;m}&= \{ \omega \in \varOmega :\text {the trajectory } \xi _t(\omega ) \text { changes values at most } m \text { times on } [0,r] \} \\&= \{ \omega \in \varOmega :t_{m} > r \}\in \mathcal {F} \ . \end{aligned} \end{aligned}$$

It follows from the construction that $\zeta$ is a composition $\zeta _r(x, \omega ) = \varPhi _r^{\xi _{\circ }(\omega )}(x)$. If $\omega \in \varOmega _{r;m}$, then the trajectory $\xi _{\circ }(\omega )$, restricted to the interval [0, *r*], belongs to $\mathfrak {H}_{r;m}(\varTheta^{*})$. By our Thm. 1, the mapping

$$\begin{aligned} \mathfrak {H}_{r;m} \ni \xi _{\circ }(\omega ) \mapsto \varPhi _r^{\xi _{\circ }(\omega )}(x) \in X \end{aligned}$$

is $(d_r, \varrho )$ continuous (so measurable). It remains to show that

$$\begin{aligned} \varOmega _{r;m} \ni \omega \mapsto \xi _{\circ }(\omega ) \in \mathfrak {H}_{r;m}(\varTheta^{*}) \end{aligned}$$

is measurable. Let $A = \{ a_0 = 0, a_1, a_2, \dots \} \subset [0,r]$ be a countable dense subset of [0, *r*]. Denote $A_k = \{ a_0, a_1, \dots , a_k \}$. Consider a closed (for the relative $d_r$ topology) ball

$$\begin{aligned} \mathcal {K}_{r;m}(\mathfrak {h}_0, \varepsilon ) = \{ \mathfrak {h} \in \mathfrak {H}_{r;m}(\varTheta^{*}) :d_r(\mathfrak {h}_0 , \mathfrak {h}) \le \varepsilon \} \ . \end{aligned}$$

By separability, we can select a countable and dense set $K = \{ \mathfrak {h}_1, \mathfrak {h}_2, \dots \}$ in $\mathcal {K}_{r;m}(\mathfrak {h}_0, \varepsilon )$. We notice that

$$\begin{aligned} \mathcal {K}_{r;m}(\mathfrak {h}_0, \varepsilon ) = \bigcap _{k=1}^{\infty }\bigcup _{j=1}^{\infty } \{ \mathfrak {g} \in \mathfrak {H}_{r;m}(\varTheta^{*}) :\mathfrak {g}(a_i) = \mathfrak {h}_j(a_i) \ \text {for \ all \ } i = 1, 2, \dots , k \}. \end{aligned}$$

Thus the trajectory $\xi _{\circ }(\omega )$, restricted to the time domain [0, *r*], belongs to $\mathcal {K}_{r;m}(\mathfrak {h}_0, \varepsilon )$ if and only if

$$\begin{aligned} \omega \in \bigcap _{k=1}^{\infty }\bigcup _{j=1}^{\infty } \{ \omega \in \varOmega _{r; m} : \xi _{a_i}(\omega ) = \mathfrak {h}_j(a_i) \ \text {for \ all \ } i = 1, 2, \ldots , k \}. \end{aligned}$$

Clearly, $\{ \omega \in \varOmega _{r; m} :\xi _{a_i}(\omega ) = \mathfrak {h}_j(a_i) \} \in \mathcal {F}\cap \varOmega _{r; m}$, as $\xi _{a_i} :\varOmega \mapsto \varTheta^{*}$ is measurable. It follows that (see Fig. 10) $\zeta _r(x, \cdot )$ is $\mathcal {F}$ measurable. To end the proof we remark that the continuity of trajectories $[0, \infty ) \ni t \mapsto \zeta _t(x, \omega ) \in X$ is a trivial consequence of our construction of $\varPhi ^{\mathfrak {h}}$. $\blacksquare$

## Footnotes

1 https://en.wikipedia.org/wiki/COVID-19_pandemic_on_Diamond_Princess.
